# Low-grade glioma harbors few CD8 T cells, which is accompanied by decreased expression of chemo-attractants, not immunogenic antigens

**DOI:** 10.1038/s41598-019-51063-6

**Published:** 2019-10-10

**Authors:** Bas Weenink, Kaspar Draaisma, Han Z. Ooi, Johan M. Kros, Peter A. E. Sillevis Smitt, Reno Debets, Pim J. French

**Affiliations:** 1 Department of Neurology, Erasmus MC Cancer Institute, Rotterdam, The Netherlands; 2 Department of Medical Oncology, Laboratory of Tumor immunology, Erasmus MC Cancer Institute, Rotterdam, The Netherlands; 3 Department of Pathology, Erasmus MC Cancer Institute, Rotterdam, The Netherlands

**Keywords:** CNS cancer, Tumour immunology

## Abstract

In multiple tumor types, prediction of response to immune therapies relates to the presence, distribution and activation state of tumor infiltrating lymphocytes (TILs). Although such therapies are, to date, unsuccessful in gliomas, little is known on the immune contexture of TILs in these tumors. We assessed whether low and high-grade glioma (LGG and HGG, grade II and IV respectively) differ with respect to number, location and tumor reactivity of TILs; as well as expression of molecules involved in the trafficking and activation of T cells. Intra-tumoral CD8 T cells were quantified by flow cytometry (LGG: n = 12; HGG: n = 8) and immunofluorescence (LGG: n = 28; HGG: n = 28). Neoantigen load and expression of Cancer Germline Antigens (CGAs) were assessed using whole exome sequencing and RNA-seq. TIL-derived DNA was sequenced and the variable domain of the TCRβ chain was classified according to IMGT nomenclature. QPCR was used to determine expression of T cell-related genes. CD8 T cell numbers were significantly lower in LGG and, in contrast to HGG, mainly remained in close vicinity to blood vessels. This was accompanied by lower expression of chemo-attractants *CXCL9*, *CXCL10* and adhesion molecule *ICAM1*. We did not observe a difference in the number of expressed neoantigens or CGAs, nor in diversity of TCR-Vβ gene usage. In summary, LGG have lower numbers of intra-tumoral CD8 T cells compared to HGG, potentially linked to decreased T cell trafficking. We have found no evidence for distinct tumor reactivity of T cells in either tumor type. The near absence of TILs in LGG suggest that, at present, checkpoint inhibitors are unlikely to have clinical efficacy in this tumor type.

## Introduction

Diffuse gliomas are the most common type of primary brain cancer in adults. Currently, they are classified based on histological and genetic features into oligodendroglioma, astrocytoma and glioblastoma^1^. Oligodendroglioma and astrocytoma are collectively known as diffuse low-grade gliomas (LGG; WHO grade II). Glioblastoma multiforme is the most aggressive glioma subtype and by definition a high-grade tumor (HGG; WHO grade IV). Approximately 80% of LGG harbor driver mutations in isocitrate dehydrogenase (*IDH*) 1 or 2 genes, while HGG are mostly *IDH* wildtype^2,3^. Although survival for diffuse gliomas differ substantially (e.g. 5-year survival for glioblastoma: 5.5%, for grade II oligodendroglioma: 81.3%), all patients eventually die from their disease^4^. New treatment options are therefore urgently required.

Immune therapies with checkpoint inhibitors (CI) have shown clinical efficacy in a number of tumor types, including melanoma, non-small cell lung cancer, renal cancer, bladder cancer, head and neck squamous cell carcinoma and non-hodgkin lymphoma^5–7^. In a fraction of patients, particularly melanoma patients, these responses are durable^8^. The best-characterized CIs include monoclonal antibodies nivolumab and pembrolizumab targeting programmed cell death protein 1 (PD-1) and ipilimumab targeting cytotoxic T-lymphocyte-associated protein 4 (CTLA-4), respectively. In gliomas, there are several phase III trials currently ongoing that test the clinical benefit of CIs, mostly in the setting of recurrent HGG. Unfortunately, an initial study has not shown overall survival benefit for recurrent HGG patients and the primary endpoint of the Checkmate 498 study on newly diagnosed patients was not met^9^. Nevertheless, some anecdotal evidence of response in hypermutated HGG has been documented^10,11^.

Absent or limited response to checkpoint inhibitors may not only be the result of a reduced antigenicity (tumor mutation burden, TMB) of the tumor, PD-L1 expression or CD8-T cell density^10,12–15^, but also to reduced egress of T cells from the bloodstream and influx into the tumor^16^. Although several of these immune evasive mechanisms have been evaluated in gliomas (gliomas for example have a low TMB and several studies showed that the antitumor immune response in HGGs is suppressed amongst others by enhanced PD-L1 expression^17–22^), most of these studies did not evaluate multiple immune parameters and/or did not evaluate potential differences between LGG and HGG. It therefore remains to be determined which of these above mentioned mechanisms potentially can contribute to the (absence of) response to checkpoint inhibition in LGG and HGG. In the present study, we have made a comprehensive inventory whether LGG and HGG differ with respect to number, location and tumor reactivity of tumor-infiltrating lymphocytes (TILs); as well as expression of molecules involved in the trafficking and activation of T cells. Collectively, our data demonstrate that LGG and HGG differ with respect to the extent of T-cell infiltration. Since checkpoint inhibitors have limited effectivity in HGG patients, the near absence of TILs in LGG suggests that such effectivity may be even more limited in this tumor type.

## Results

### LGG shows low numbers of T cells that are located perivascularly

**Figure 1:**
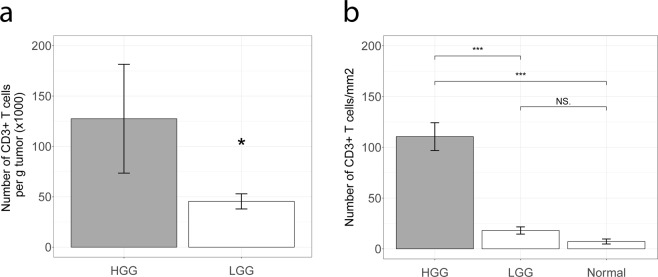
T cells are less abundant in LGG versus HGG. (**a**) Single cell suspensions of HGG (n = 8) and LGG (n = 12) were used to enumerate CD3 + T cells using flow cytometry. Gating strategy is shown in Fig. S1. Data are represented as mean ± SEM, p < 0.05, Mann-Whitney U test. (**b**) T cells were quantified on an independent set of HGG (n = 28) and LGG (n = 28) using CD3 immune stainings. Mann-Whitney U test. ***P < 0.001, *P < 0.05, NS = not significant.

We first assessed whether LGG and HGG differ with respect to the number of intra-tumoral T cells. To this end, we have used two techniques. First, using flow cytometry (LGG: n = 12; HGG: n = 8), we found a ~2.5 fold decrease in the number of T cells in LGG when compared to HGG (Fig. 1a). Second, we quantified T cells on an independent set of tumors with immune stainings (LGG: n = 28; HGG: n = 28). Again, we observed that T cell numbers were decreased in LGG by approximately 5 fold when compared to HGG (Figs 1b, S1). Normal brain tissues (n = 4) showed virtually no presence of intra-tumoral T cells.

**Figure 2:**
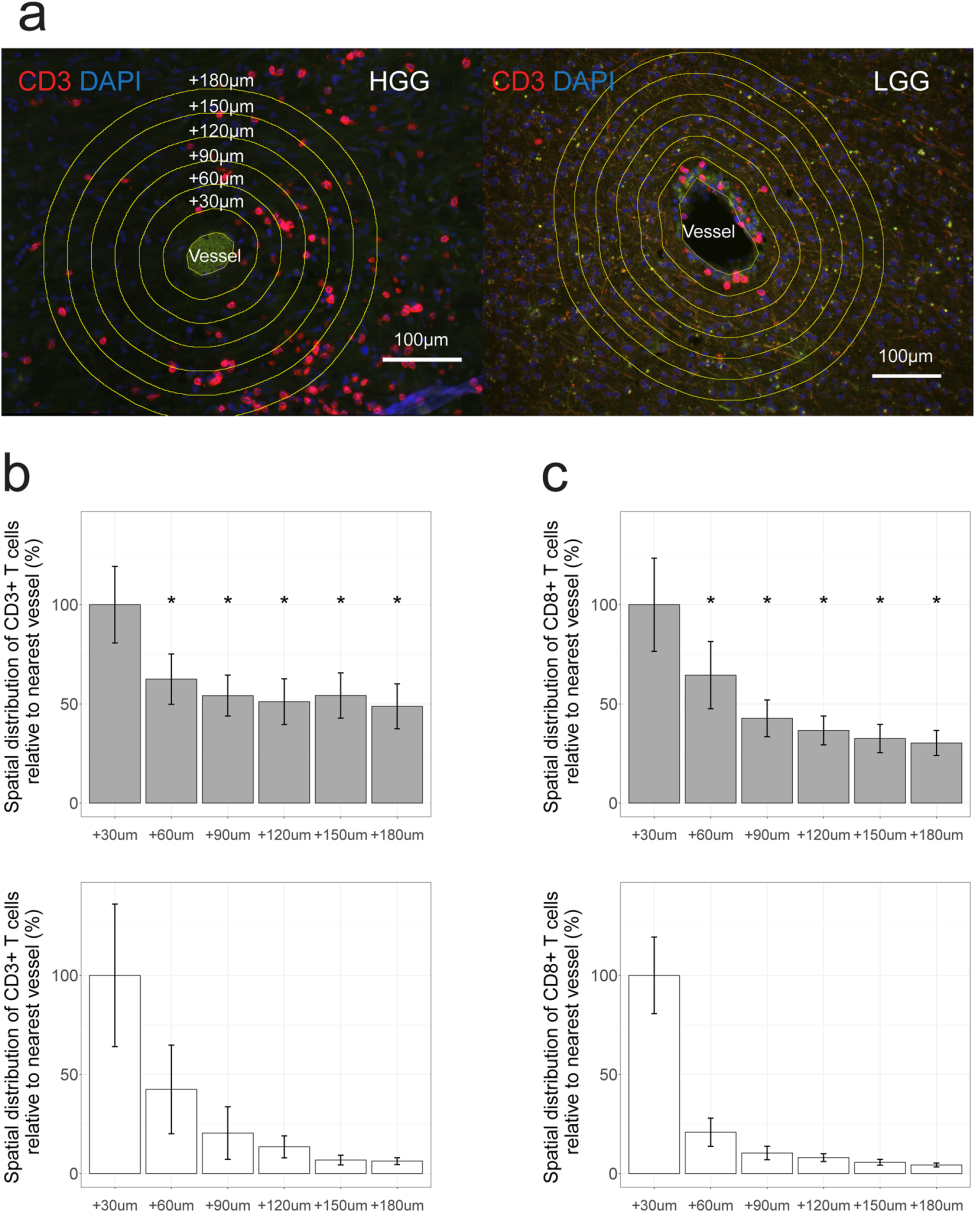
T cells are retained to blood vessels in LGG compared to HGG. Spatial distribution of T cells in HGG (n = 28) and LGG (n = 28) was determined by CD3 and CD8 immune stainings. (**a**) T cells were quantified in consecutive perimeters of 30 µm (30–180 µm) starting from the border of vessels from different tumor regions (left panel; representative HGG example, right panel; representative LGG example, stained for CD3). Vessels were identified using autofluorescence and are positive for CD31 (see also Fig. S2). Magnification is 20×. Scale bar is 100 µm. (**b**) Spatial distribution of CD3 and (**c**) CD8 T cells in HGG (top) vs LGG (bottom). Mean number of T cells within first perimeter is set at 100%. Values for each perimeter are compared between HGG and LGG, Mann-Whitney U test. *P < 0.01.

Besides T cell numbers, we next used our immune stainings to assess the location of T cells in both tumor types. We noted that T cells predominantly localize in the vicinity of blood vessels (Fig. S2). Interestingly, CD3 and CD8 T cells showed a deeper invasion into the tumor tissue in HGG versus LGG. In fact, in LGG, T cells were predominantly located perivascularly (Fig. 2a–c), and T cells were only rarely identified within more distant vessel perimeters. No difference in vessel size was observed between both tumor types (Fig. S2). Taken together, our data show that in LGG there are fewer T cells than in HGG and they invade less deep into the tumor parenchyma.

We also evaluated whether T cell numbers are related to *IDH* mutation status as several reports indicated a causal role for mutated *IDH* (and D2HG accumulation) in immune suppression^23,24^. *IDH1/2* mutation status was available for 35 (100%) LGG samples and 21 (68%) HGG samples used for TIL immune stainings and flow cytometry. In our dataset, only one LGG was *IDH* wildtype, but nevertheless showed low TIL counts corresponding to nearly all *IDH* mutant LGG samples (<40 T cells/mm^2^). *IDH1* mutations were observed in two HGG, and these samples indeed had relatively low T cell numbers, comparable to LGG (<40 T cells/mm^2^). However, with these limited IDH wildtype (LGG) or IDH mutant (HGG) sample numbers, we cannot conclude on a potential association between IDH status and T cell abundance.

### LGG and HGG do not show differential expression of neo-antigens nor cancer germline antigens

**Figure 3:**
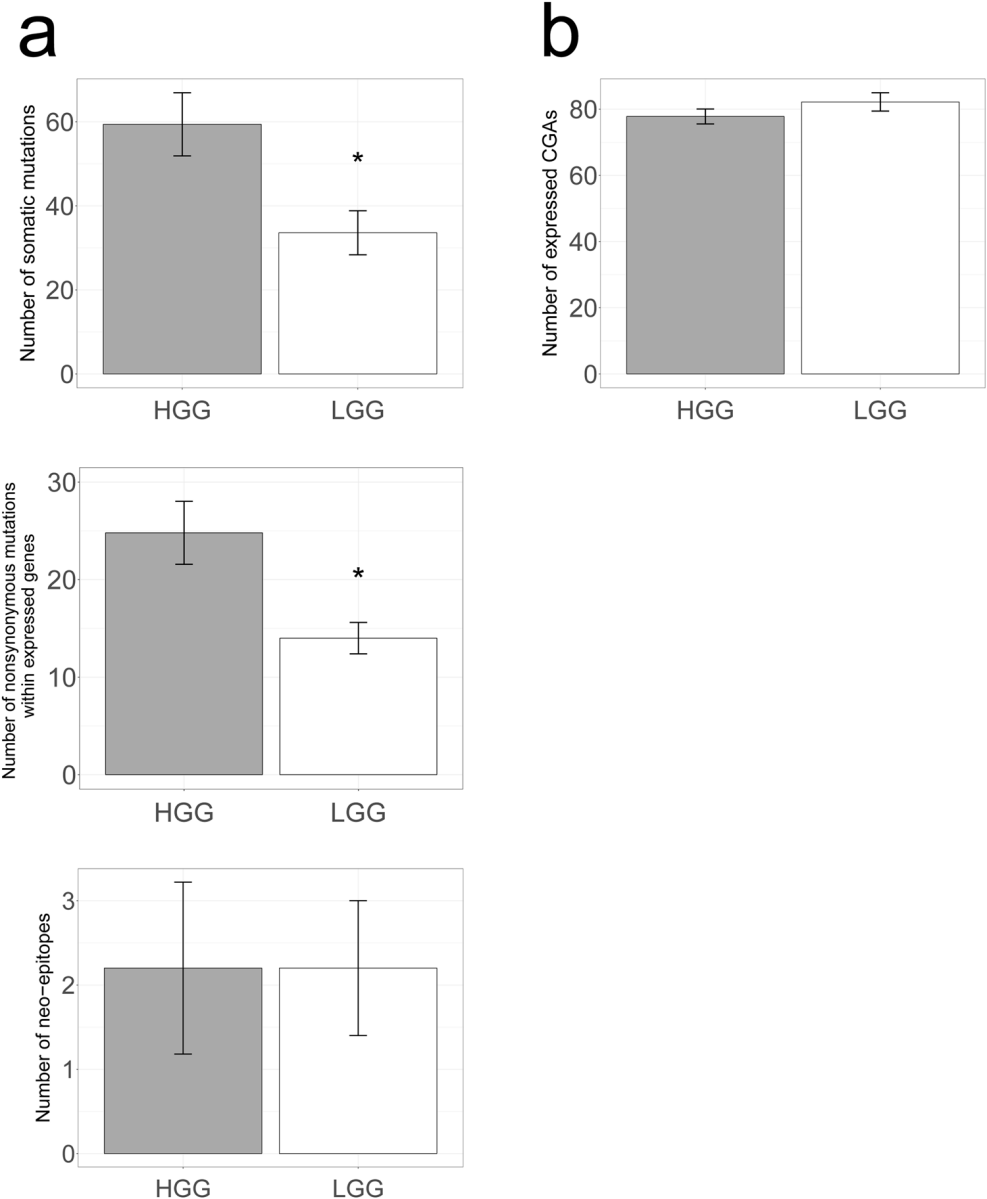
Neo-antigens nor CGAs are differentially expressed in LGG versus HGG. (**a**) The total number of mutations in coding regions in HGG (n = 5) and LGG (n = 5) shows a higher mutational load in HGG (top). This difference is also observed when selecting for nonsynonymous mutations that are expressed (middle). The number of predicted neo-antigens were however very low and did not differ between LGG and HGG (bottom). See materials and methods for details. (**b**) Number of expressed CGAs was similar in LGG and HGG samples. Mann-Whitney U test. *P < 0.05.

Since the observed differences in quantity and location of CD8 T cells could be due to different levels in antigenicity, we have determined the quantity of: (i) neo-antigens that may arise from expressed somatic mutations and (ii) cancer germline antigens (CGAs) that may arise from loss of epigenetic silencing. Both can be exclusively expressed in malignant but not healthy tissues. On average, LGG harbor twice as few coding non-synonymous mutations compared to HGG (Fig. 3a, 1^st^ panel: 34 ± 12 and 59 ± 17) which is in concordance to previously published reports^17^. We find that less than half of these mutations are expressed (on average 14 ± 4 and 25 ± 7 in LGG and HGG, Fig. 3a, 2^nd^ panel). Importantly, on average only two of these expressed mutations per tumor scored sufficiently high according to MHC class I binding, proteasomal C terminal cleavage and TAP transport efficiency to be considered a neo-epitope. This low number was similar in both LGG and HGG (Fig. 3a, 3rd panel, Fig. S3 and Table S3). When analyzing expression of CGAs (all those listed in the CT Database, available at http://www.cta.lncc.br/), we found no difference when comparing between LGG and HGG (Figs 3b, and S3). These results indicate that differences in T cell numbers and locations are unlikely caused by differences in levels of immunogenic antigens, such as neo-antigens and CGAs.

### LGG and HGG harbor an equally diverse TCR-Vβ repertoire

**Figure 4:**
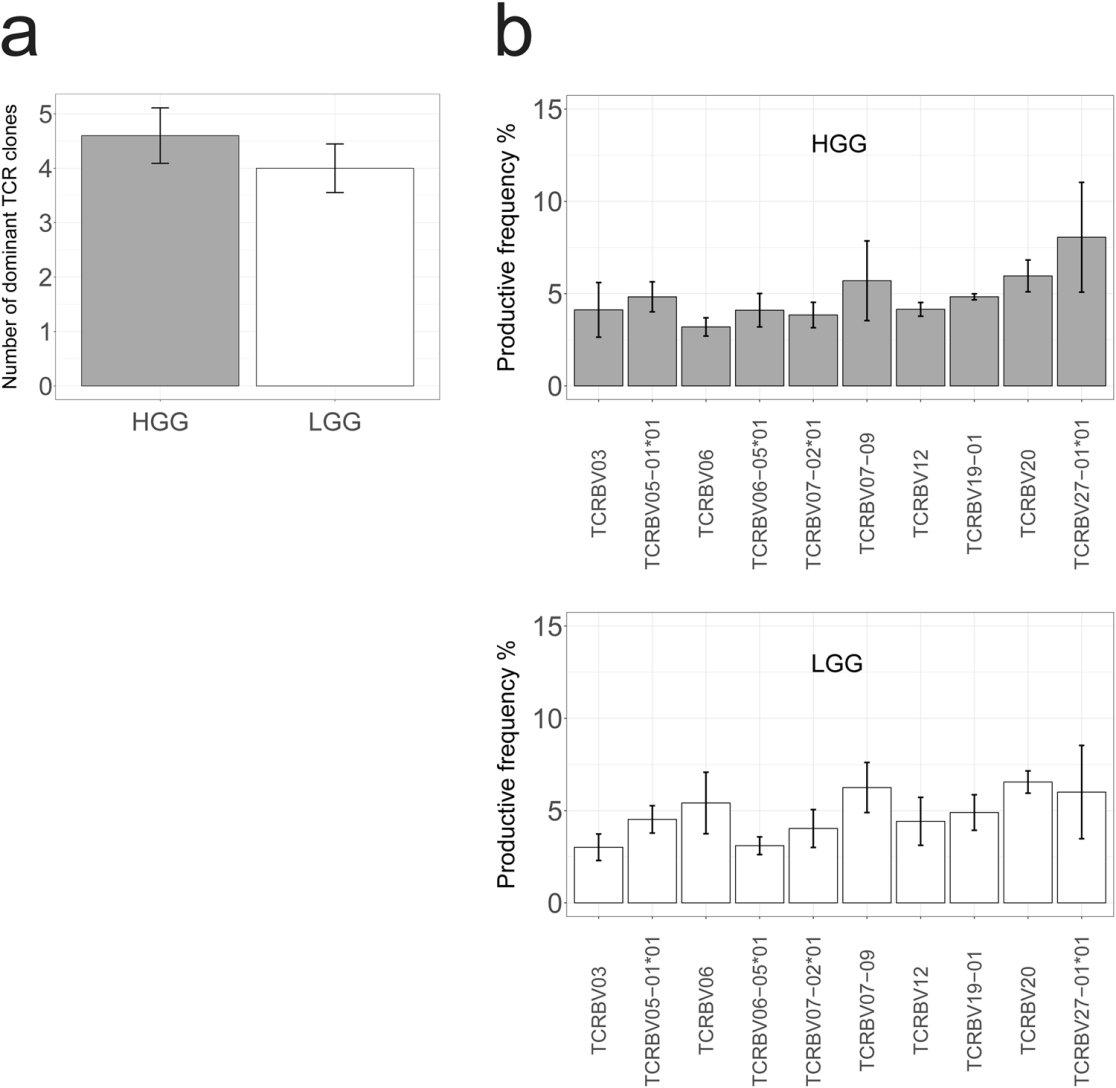
TCR repertoire appears diverse in HGG and LGG, showing no difference in Vβ dominance. TIL-derived DNA of HGG (n = 5) and LGG (n = 5) was sequenced for the CDR3 region of the TCRβ chain. (**a**) HGG and LGG do not differ in quantity of dominant TCR clones. The average number of dominant TCR clones is determined by the number of TCR-Vβ genes representing 30% of all Vβ genes and presented per patient sample. (**b**) HGG and LGG do not differ in quality of dominant TCR clones. The quality of TCR clones is determined by the gene-usage of the 10 most frequent TCR-Vβ genes and presented for HGG (top) or LGG (bottom). Productive frequency (%) is the fraction of unique TCR-Vβ reads which do not contain a premature stop-codon. Mann-Whitney U test.

Another angle to assess differences in antigenicity between HGG and LGG is the breadth of the TCR-Vβ repertoire of intra-tumoral T cells. To this end, we have sequenced the TCRβ CDR3 region of T cells present in the same tumors that were used for exome and RNA sequencing. Our data show that LGG and HGG do neither differ in quantity (Fig. 4a), nor quality (diversity; the number of unique and productive TCR-Vβ reads that represent the top 30% of the total number of TCR-Vβ reads), nor quality (convergence; the abundance of the 10 most frequent TCR-Vβ reads) of dominant TCR clones (Figs 4b, S4). Of note, in HGG patient #2 and LGG patients #1 and #5, we observed only 3 highly abundant TCR clones and thus a clear narrowing down of the TCR-Vβ repertoire, which may be indicative of an active T cell response. Since the TCR-Vβ repertoire appears equally diverse in LGG and HGG, our results confirm the notion that the observed differences in T cell numbers and location between LGG and HGG are not driven by differences in tumor antigenicity. However, neo-epitope identification should be interpreted with caution as these tools may have a significant false negative rate. It is also noteworthy that besides the presence of neo-epitopes, antigen recognition by T cells depends on other variables, such as antigen processing, immunogenicity of epitope, and/or MHC expression. With respect to the latter, class II MHC-restricted epitopes have not been assessed in the current study.

### LGG demonstrates decreased expression of T cell chemo-attractants and adhesion molecules

**Figure 5:**
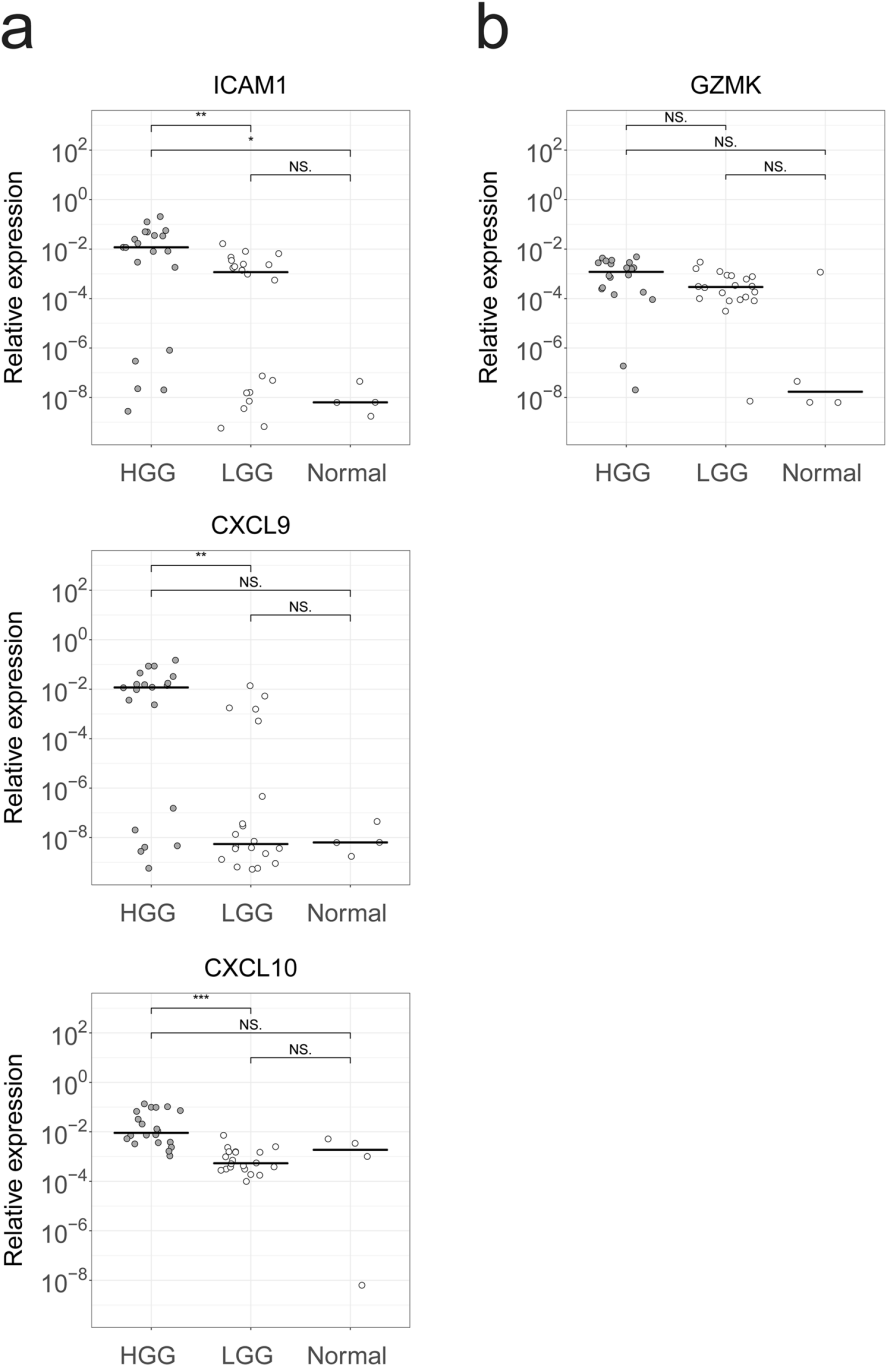
Decreased gene-expression of T cell trafficking molecules in LGG versus HGG. RT-qPCR was performed for HGG (n = 20) and LGG (n = 20) to determine gene expression levels of (**a**) the chemo-attractants CXCL9 and CXCL10, adhesion molecule ICAM1 and (**b**) the T cell effector molecule GZMK. Each dot represents an individual sample. Relative expression levels (2^−ΔCt^**)** are shown according to log scale. Black bars show the median value. Expression levels of HGG and LGG are compared using the Mann-Whitney U test and p-values are FDR-corrected for multiple testing. ***P < 0.001, **P < 0.01, *P < 0.05, NS = not significant.

Lastly, we analyzed the expression of genes that correspond to T cell recruitment (HGG: n = 20, LGG: n = 20, Fig. 5a,b). In line with the observed decrease in T cell numbers, T cell adhesion molecule *ICAM1* and chemo-attractants *CXCL9* and *CXCL10* were expressed at lower levels in LGG compared to HGG. No correlation could be found between expression level of these factors and intratumoral CD8 T cell numbers (data not shown). Expression of CD8 T cell effector molecule *GZMK* was also decreased, yet this difference did not reach statistical significance. We confirmed these observations in TCGA RNA-seq data of LGG and HGG. Table 1 shows an overview of adhesion molecules, chemo-attractants and molecules associated with CD8 T cells and fold differences in their expression when comparing LGG to HGG. Normal brain tissues (n = 4) did not express any of above-mentioned markers except *CXCL10*, which showed mRNA levels similar to LGG samples.

**Table 1:** Expression of T cell recruitment genes in LGG and HGG.

Chemo-attractants	LGG (mean ± SD)	HGG (mean ± SD)	Fold change	P-value
*CXCL10*	4.7 ± 2.4	8.6 ± 2.0	0.6	2.E-43
*CXCL11*	3.6 ± 2.0	6.4 ± 2.1	0.6	2.E-28
*CXCL9*	4.5 ± 2.2	7.0 ± 1.9	0.7	7.E-23
*CCL5*	6.1 ± 1.8	7.6 ± 1.2	0.8	4.E-17
*CCL2*	8.9 ± 2.0	10.6 ± 1.7	0.8	1.E-14
*CCL3*	9.1 ± 2.4	7.6 ± 1.9	1.2	4.E-10
*CCL7*	0.3 ± 1.1	1.6 ± 2.2	0.2	9.E-10
*CCL4*	8.4 ± 2.5	7.2 ± 1.7	1.2	5.E-07
*CCL1*	0.4 ± 1.0	0.1 ± 0.4	5.3	2.E-05
*CXCL3*	4.7 ± 1.7	5.4 ± 2.3	0.9	4.E-03
*CXCL4*	1.6 ± 1.6	1.5 ± 1.6	1.1	4.E-01
**Cell adhesion, motility and movement**	**LGG (mean ± SD)**	**HGG (mean ± SD)**	**Fold change**	**P-value**
*ANXA2*	10.6 ± 1.1	13.1 ± 1.0	0.8	2.E-59
*LGALS1*	11.5 ± 0.9	13.4 ± 0.9	0.9	2.E-54
*LGALS3*	9.7 ± 0.9	12.3 ± 1.4	0.8	8.E-48
*TSPAN24*	11.6 ± 0.6	13.0 ± 0.9	0.9	6.E-38
*ANXA5*	13.3 ± 0.7	14.6 ± 0.9	0.9	2.E-34
*TSPAN30*	13.9 ± 0.5	15.0 ± 0.8	0.9	1.E-29
*ICAM1*	9.2 ± 1.3	10.5 ± 1.3	0.9	1.E-15
*PECAM1*	9.7 ± 0.8	10.4 ± 0.7	0.9	5.E-13
*ITGB2*	11.5 ± 1.4	12.3 ± 1.1	0.9	5.E-07
*TSPAN10*	3.7 ± 1.5	4.3 ± 1.5	0.9	2.E-04
*ITGAL*	8.2 ± 1.6	8.7 ± 1.3	0.9	6.E-04
*TSPAN28*	15.3 ± 0.6	15.1 ± 0.7	1.0	3.E-03
*VCAM1*	10.7 ± 2.0	11.1 ± 1.4	1.0	2.E-02
*ICAM2*	9.0 ± 0.7	8.8 ± 0.8	1.0	5.E-02
**CD8 T cells**	**LGG (mean ± SD)**	**HGG (mean ± SD)**	**Fold change**	**P-value**
*ICOS*	1.0 ± 1.5	2.4 ± 1.7	0.4	2.E-13
*GZMK*	2.5 ± 2.1	4.3 ± 2.1	0.6	1.E-12
*CD8A*	5.1 ± 1.6	6.2 ± 1.4	0.8	2.E-10

TCGA gene expression data of molecularly defined LGG and HGG cases (LGG: n = 232, HGG: n = 246) were used from the University of California Santa Cruz Cancer Genome Browser (available at http://www.cbioportal.org/). LGG and HGG cases were compared for expression levels of differentially expressed C, CC, CXC and CX3C chemo-attractants, adhesion molecules (including integrins, annexins, galectins, tetraspanins) and genes associated with CD8 T cell activation. Mean expression values ± SD for each gene are shown as log2 (FKPM) and LGG vs HGG are tested using T tests. P-values are FDR-corrected for multiple testing. Fold change was calculated by dividing LGG mean expression value by HGG mean expression value. Genes shown in bold show significantly lower expression in LGG.

## Discussion

In this study, we have investigated whether LGG and HGG differ with respect to number and location of T cells. We observed that CD8 T cells are less abundant and are more closely located to blood vessels in LGG compared to HGG. We show that this T cell exclusion is unlikely caused by differential tumor reactivity of T cells nor levels of neo-epitopes or CGAs, but that these differences are accompanied by differential expression of T cell chemo-attractants and adhesion molecules.

Previous studies also reported reduced CD8 T cell numbers in human LGG compared to HGG^23,25,26^. Most LGG harbor mutations in the *IDH1* gene and there is recent evidence for a causal role of mutant IDH1 activity in immune suppression. For example, in a murine model for LGG, the introduction of mutant *IDH1* or treatment with 2HG reduced protein levels of CXCL10 likely through decreased production of STAT1^23^, and suppressed the accumulation of T cells at tumor sites. A different study showed that 2HG reduced proliferation of human T cells cultured *in vitro*^24^. However, we could not conclude on a potential relationship between IDH status and T cell abundance, since either IDH wildtype or IDH mutant sample numbers were too small for LGG and HGG, respectively.

Although T cells are clearly present in HGG, multiple immune suppressive mechanisms have been shown to attenuate an effective antitumor immune response in this tumor^18,19^. The co-presence and function of immune suppressive cells like regulatory T cells (Tregs), tumor-associated macrophages (TAMs) and myeloid-derived suppressor cells (MDSCs) results in strong dampening of T cell effector function in the HGG microenvironment. Besides the immune suppressive cells, also up-regulated expression of immune or metabolic checkpoint inhibitors (i.e., PD-1 and IDO1, respectively) dampens T cell effector function in HGG^18–21^. A recent study analyzed PD-1+ T cell infiltration and also PD-L1 tumor cell expression in 57 *IDH* mutant and 117 *IDH* wildtype gliomas and found that *IDH* wildtype HGG gliomas display more prominent PD-1+ T cells and higher PD-L1 expression when compared to *IDH* mutant LGG cases^25^. Garber and colleagues also showed a positive correlation between PD-1+ T cells and high but not low tumor grade^27^. In addition, several other studies have confirmed high PD-L1 expression on tumor cells in HGG^28–30^, yet there are large differences in extent of PD-L1 positivity between studies, which may relate to differences in primary antibody used.

There is some preclinical evidence for the effectivity of (combination) checkpoint inhibition in glioma^31,32^, which compelled the clinical testing of inhibitors targeting CTLA-4 and PD-1 in (recurrent) HGG patients. However, preliminary results demonstrated a failure of nivolumab to prolong overall survival of patients with recurrent HGG, and the primary endpoint was not met in newly diagnosed, *MGMT-*promoter unmethylated patients treated with nivolumab in combination with temozolomide and radiotherapy^9^. Studies on melanoma and non-small-cell lung carcinoma have shown that response to checkpoint inhibitors is associated with a high mutational load^12,13^. Indeed, nivolumab treatment of 2 patients with hypermutant HGG resulting from germline biallelic mismatch repair deficiency (BMMRD) resulted in clinical responses^10^. In most HGG and LGG mutational burden is more than an order of magnitude lower than in melanomas and lung adenocarcinomas^17^. Our data, using whole exome sequencing, show that HGG on average harbor only ~60 coding mutations and LGG half this amount, which is in line with data from other studies^21,33^. This number is significantly lower than the ~150 non-synonymous mutations within expressed genes that are estimated to be required, on average, to establish neo-epitopes that can potentially be recognized by autologous T cells. Although ~150 non-synonymous mutations present in expressed genes within a single tumor generally enable the detection of neo-antigens^34^, it is important to realize that a single mutation may already be sufficient to mediate an anti-tumor response. When assessing neo-epitope expression, we predicted presence of only a very few (up to 2) in both LGG and HGG, and expression levels were very low. With respect to CGAs, approximately 80 were expressed in both tumor types, however again at low levels. Of note, it should be considered that CGAs may not be presented in each patient (tumor) due to HLA restriction of CGA epitopes. Although expression analysis was performed on a rather small number of samples (n = 5 for both tumor types), neo-epitope and CGA expression values demonstrated limited variation. Despite low expression of above-mentioned antigens, we did observe a clear narrowing of the TCR repertoire in 30% of tumor samples, similar for both LGG and HGG, which suggests that intra-tumoral T cells have been enriched for reactivity against tumor tissue. Indeed, there is preclinical evidence for the immunogenicity of a number of neo-epitopes arising from hallmark glioma mutations, including EGFRvIII (an intragenic deletion of exons 2–7 of the epidermal growth factor receptor), IDH1^R132H^ and H3.3^K27M ^^35–37^. Future studies will have to determine whether LGG and HGG are inherently sufficiently immunogenic for CI to be effective as a single therapy.

Based on our results, we speculate that low numbers and perivascular location of T cells in LGG is at least partly due to hampered T cell trafficking into tumor parenchyma. Microvascular proliferation is a specific histological hallmark for HGG and the absence of this phenomenon in LGG translates to lower abundance of endothelium and may consequently also result in lower levels of T cell trafficking molecules. Accordingly, we observed low expression of the adhesion molecule *ICAM1* and the chemo-attractants *CXCL9* and *CXCL10* in LGG, molecules that have earlier been reported to be related to T cell exclusion^38^. This could be confirmed in TCGA RNA-seq data, where a general downregulation of cell adhesion, motility and movement molecules, and chemo-attractants could be seen in LGG compared to HGG. The impact of chemo-attractants on the observed differences in T cell numbers still remains speculative and future functional experiments should be performed to demonstrate causality. Many other factors could also contribute to these observations, including exhaustion of T cells, presence of immunosuppressive cells and tumor-derived immunosuppressive molecules (e.g. 2HG and immune checkpoint ligands)^21,26^. Of note, LGG showed a two-fold decrease in expression of chemokine receptor *CXCR3* compared to HGG (data not shown). CXCR3+ T cells exhibit increased recruitment upon CXCR3 binding to ligands CXCL9 and CXCL10. We also found that *GZMK* is downregulated in LGG; it is expressed by CD8 T cells and this observation is in line with decreased CD8 T cell numbers in LGG. Although our data suggests correlation but does not directly prove causality, the reduced intra-tumoral CD8 T cell numbers in LGG may be explained by the aberrant expression of genes that are involved in T cell recruitment.

Taken together, the low antigenicity (neo-antigen repertoire and CGA expression) of gliomas helps understand the thus-far limited clinical efficacy of checkpoint inhibitors in glioma patients. Moreover, the low abundance and perivascular location of CD8 T cells in LGG suggest such therapies, at present, will have little effect on LGG patients.

## Methods

### Tumor samples

HGG (grade IV) and LGG (grade II) samples were randomly selected from the Erasmus MC tumor archive based on histological and molecular WHO 2016 criteria. IDH1 status was determined for diagnostic purposes by next generation targeted resequencing or immunohistochemistry for most cases^39,40^. We performed sanger sequencing on DNA isolated from FFPE tumor tissue samples in case of unknown IDH1 mutation status^41^. For immune stainings, samples with significant areas of necrosis were avoided. Samples for whole exome, RNA and TCR-seq were selected for tumor cell percentages higher than 70%, as determined by a dedicated neuropathologist (J.M.K.). TIL samples (see below) were derived from tumor biopsies or tumor tissue collected with the Cavitron Ultrasonic Surgical Aspirator (CUSA). DNA isolated from healthy control PBMC was available at the Erasmus MC department of Clinical Chemistry. All patients provided written informed consent according to national and local regulations for correlative tissue studies. All experimental protocols were approved by the Erasmus MC Medical and Ethical Review Committee. The methods were carried out in accordance with the relevant guidelines and regulations. Patient characteristics are listed in Tables S1 and S2.

### TIL suspensions and flow cytometry

Tumors were cut in small pieces. Red blood cell lysis buffer was added to eliminate erythrocytes, and tissues were enzymatically dissociated using 1 mg/ml collagenase and 1 mg/ml DNAse I at 37 °C for 1 hour. Dispersed tissue was passed through a 70 μm cell strainer and 0.1 mM EDTA was added to inhibit the remaining enzymatic activity of collagenase and DNAse I. Cells were stained using a mix of 5 µl PE-conjugated mouse anti-human CD45 (DAKO (Agilent Technologies), 2.5 µl APC-conjugated mouse anti-human CD3 and 1 µl 7-AAD (both from BD Biosciences (Vianen, the Netherlands)) in a final volume of 50 µl at 4 °C for 30 minutes. Cells were then washed in PBS and fixed in 1% paraformaldehyde for 5 minutes. Live T cells (7AAD−, CD14−, CD3+) were quantified on a FACS Canto (BD Biosciences) and flow cytometric data was analyzed using FlowJo V10. To correct for variation in tumor size, T cells were enumerated per gram of tumor tissue.

### Immunofluorescence and quantitation of T cell numbers

Formalin-fixed, paraffin-embedded tumor samples were cut into 4 µm sections. After deparaffinization with xylene, antigen retrieval was performed in the microwave for 20 minutes. Non-specific binding was blocked by adding 5%BSA/PBS for 30 minutes. The sections were washed in PBS and incubated for 1 hour with one of the following mouse anti-human primary antibodies: anti-CD3 clone F7.2.38, 1:100; anti-CD8 clone C8/144B, 1:200, anti-CD31 clone JC70A, 1:30 (all from Agilent Technologies, Amstelveen, the Netherlands); and, subsequently, with goat anti-mouse biotinylated, 1:200 (Agilent Technologies). A tertiary step was performed with Avidin-Cy3, 1:100 (Sanbio, Uden, the Netherlands) for 1 hour at room temperature. Slides were mounted with Vectashield Hard Set Antifade Mounting Medium with DAPI (Vector (Brunschwig chemie, Amsterdam, the Netherlands). Human normal tonsil was used as positive control tissue. Widefield fluorescent images were acquired using the LSM 700 Confocal (Zeiss, Breda, the Netherlands) in four random tumor areas. T cell counting was performed blinded to tumor subtype. Intra-tumoral vessels were identified using autofluorescence. 3 × 3 Tile images were acquired using a 20x objective. Per tumor, CD3 and CD8 T cells in the vicinity of at least 5 vessels from different tumor regions were quantified using ImageJ and custom R scripts. To this end, starting from the outside of each vessel, T cells were enumerated along concentric perimeters up to 180 µm (with 30 µm increments) and displayed per perimeter area (mm^2^).

### Antigen load

For whole exome and RNA sequencing, nucleic acids were isolated from 5 HGG and 5 LGG (see Table S2) using an AllPrep DNA/RNA Mini Kit (Qiagen, Venlo, the Netherlands) according to the manufacturer’s instructions. Exome capture was performed using the Nimblegen SeqCap EZ MedExome kit (Roche, Woerden, the Netherlands) which captures 47 Mb of exonic regions. Paired-end 2 × 100 bp sequencing was performed on HiSeq2000 systems using the TruSeq V3 chemistry (Illumina, Eindhoven, the Netherlands). For RNA sequencing, poly-A selected RNA libraries were prepared using the TruSeq Total RNA-seq library protocol (Illumina), and the resulting libraries were sequenced on an HiSeq2500 using 50 bp paired-end reads. A summary of the bioinformatic pipeline used to subsequently process our whole exome and RNA sequencing raw data is available in Supplementary methods. Using a custom R script and the Ensembl API, coding variants obtained from whole exome sequencing were edited into cDNA sequence and subsequently translated into protein sequence, after which 17 aa protein sequences (with mutated residue at middle position) were used to perform epitope predictions using NetCTLpan^42^. To determine MHC class I restriction for each patient, 4 digit typing on RNA-seq reads was performed using seq2hla^43^. Potential neo-epitopes (9 mers) were identified, selecting those epitopes that (1) contain the mutated amino acid; (2) were derived from a gene with expression (FPKM) values >0; (3) have a score >0.5 for peptide MHC class I binding, proteasomal C terminal cleavage and TAP transport efficiency. All SNVs are listed in Table S4.

### TCR-Vβ sequencing

Mononuclear cells were isolated from TIL suspensions using Percoll density gradient centrifugation, and viable T cells were quantified using flow cytometry. DNA isolation was performed using a QIAamp DNA Blood Mini Kit (Qiagen) according to the manufacturer’s instructions. Amplification and sequencing of TCRβ CDR3 was performed using the immunoSEQ Platform (Adaptive Biotechnologies, Seattle, WA).

### Real-time qPCR of immune-related genes

Four 10 µm sections of formalin-fixed, paraffin-embedded tissue were deparaffinized in xylene and dehydrated in 100% ethanol. RNA was extracted using the RNeasy FFPE Kit (Qiagen) according to the manufacturer’s instructions. Reverse transcription was performed with 40 ng of total RNA using the TaqMan PreAmp Master Mix Kit and 200IU SuperScript III Reverse Transcriptase (both from Fisher Scientific, Landsmeer, the Netherlands). QPCR was performed using a MasterMix Plus without UNG (Eurogentec, Maastricht, the Netherlands), 450 nM of each primer, 125 nM of probe, 2 µl of the preamplification mix in a final reaction volume of 20 µl. Reactions were performed and analyzed using a Stratagene Mx3005P thermocycler and Stratagene Mx3005P software (Agilent Technologies). Cycling conditions were: 50 °C for 2 minutes, 95 °C for 10 minutes, 40 cycles of 92 °C for 15 sec and 60 °C for 1 min. GAPDH was used as a housekeeping gene in all gene expression assays.

### Statistics

Statistical analysis was performed using RStudio software and statistical methods used are specified in the figure legends for each experiment. We considered differences significant when P < 0.05. Data are represented as mean ± SEM unless otherwise stated.

## Supplementary information

Supplementary methods figures and tables

## Data Availability

All data generated or analysed during this study are included in this article (and its Supplementary Information files).
