# Supplementary material for: Low-grade glioma harbors few CD8 T cells, which is accompanied by decreased expression of chemo-attractants, not immunogenic antigens
Source: Sci Rep. 2019 Oct 10;9:14643. doi: 10.1038/s41598-019-51063-6 (PMC6787014; doi:10.1038/s41598-019-51063-6)
Supplement: Supplementary file 1 — Supplementary methods figures and tables [file 41598_2019_51063_MOESM1_ESM.pdf]

Article title: Low-grade glioma harbors few CD8 T cells, which is accompanied by decreased expression of chemo-attractants, not immunogenic antigens

Author names: Bas Weenink, Kaspar Draaisma, Han Z. Ooi, Johan M. Kros, Peter A. E. Sillevis Smitt, Reno Debets, Pim J. French

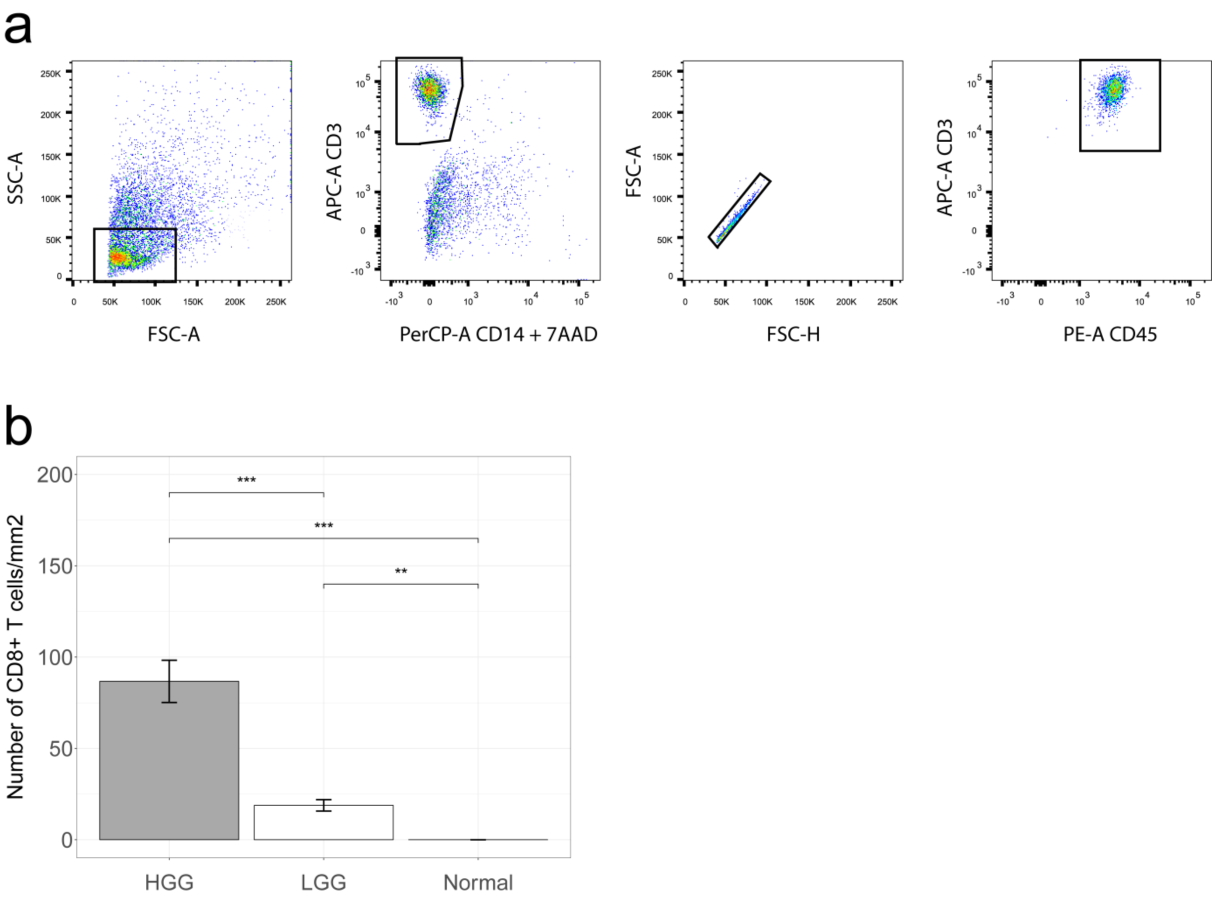

Fig. S1.

Quantification of T cells in LGG versus HGG. (A) Gating strategy used in flow cytometry analysis to detect CD3 T cells in HGG (representative example shown) and LGG. Lymphocyte populations were gated on forward scatter (FSC)/side scatter (SSC). Live T cells (7AAD-, CD14- , CD3+) were further gated and doublets were excluded using FSC-Height (FSC-H) and FSC-Area (FSC-A). Finally, T cell counts were enumerated. (B) T cells were quantified on an independent set of HGG (n=28) and LGG (n=28) using CD8 immune stainings. Mann-Whitney U test. *** = P < 0.001, ** = P < 0.01

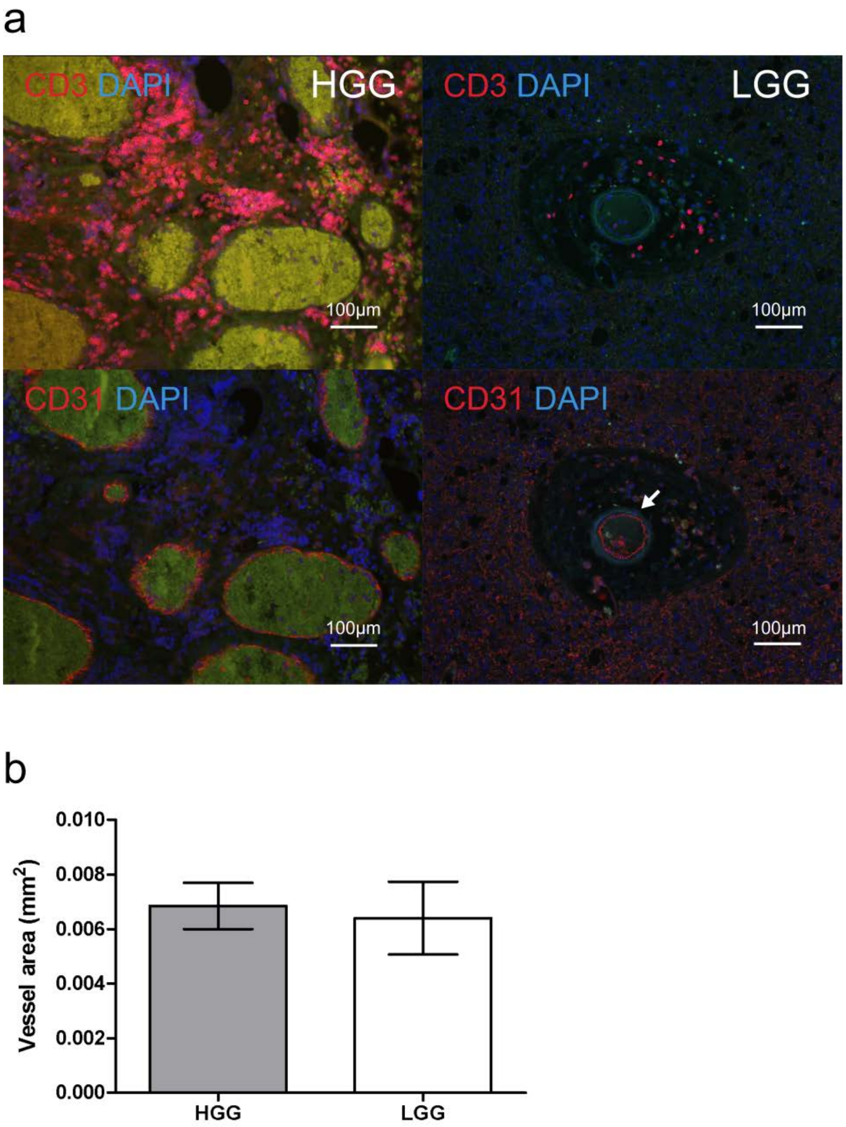

Fig. S2.

LGG and HGG do not differ with respect to luminal size of blood vessels. (A) Consecutive tissue slides of a representative LGG and HGG sample (right and left) were stained for T cells (CD3) and endothelial cells (CD31). T cells were predominantly found near blood vessels in both subtypes. Magnification is 20x. Scale bar is 100µm. (B) Five vessels from different tumor regions were identified in a set of HGG (n=10) and LGG (n=10) using CD31 immune stainings and subsequently, assessed for vessel areas using ImageJ software. No significant differences in luminal blood vessel sizes were observed between both tumor types. Mann-Whitney U test

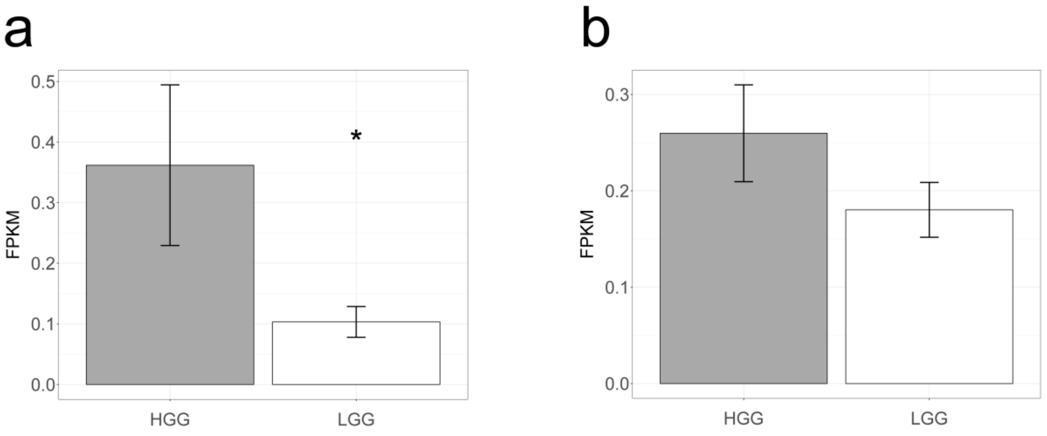

Fig. S3.

Expression levels of neo-epitopes and CGAs are low in LGG and HGG. (A) Expression levels of neo-epitopes in LGG vs HGG (both n=5, same tumors as in Figure 3) were assessed using RNA-seq. (B) Expression levels for CGAs in same samples. Values in FPKM (Fragments Per Kilobase Million) are shown on the y-axis. Mann-Whitney U test. * = $P < 0.05$

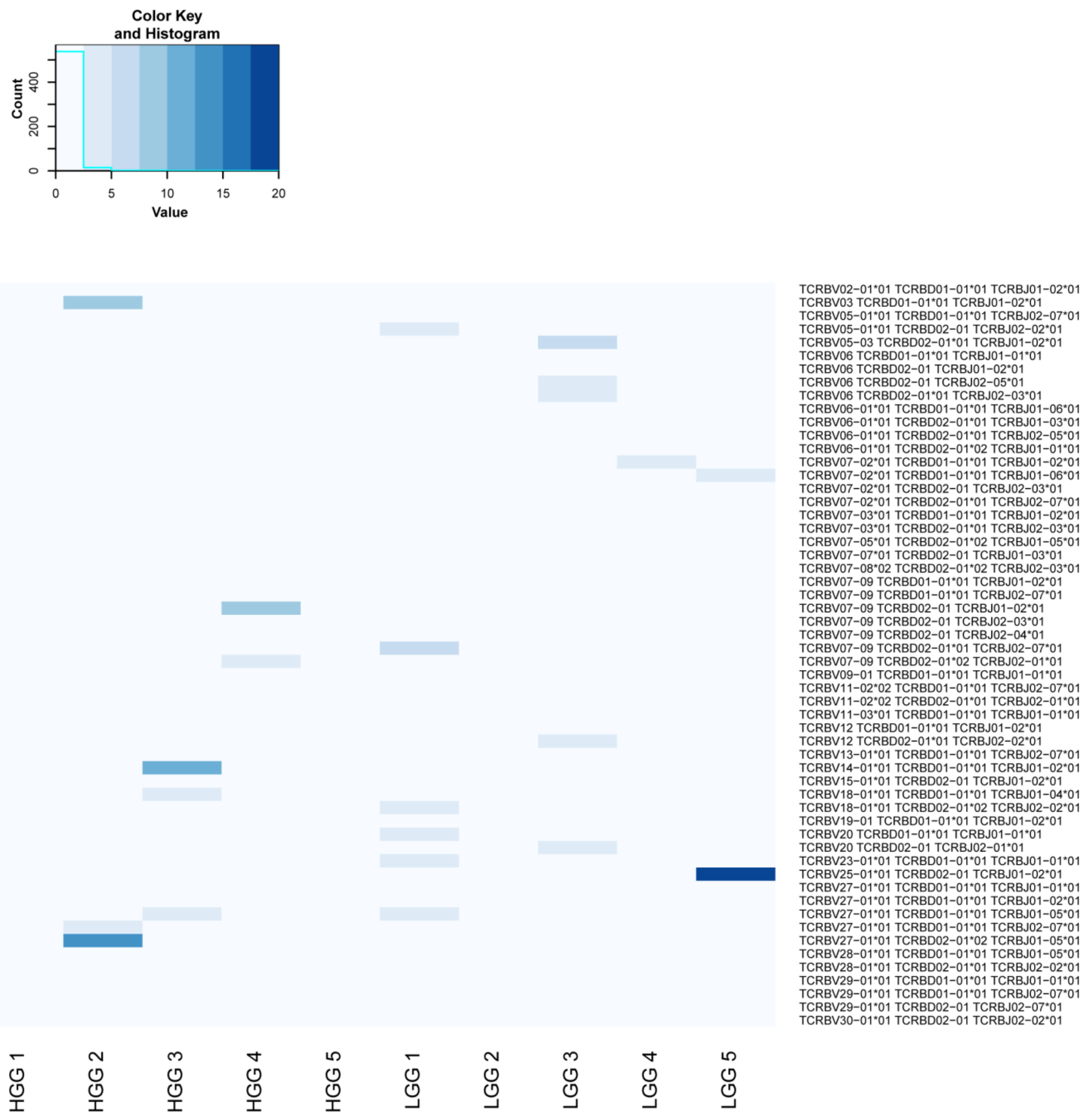

Fig. S4.

LGG and HGG do not show distinct TCR-VDJβ gene usage. TIL-derived DNA of LGG and HGG (both n=5, same tumors as in Figure 4) was sequenced for the CDR3 region of the TCRβ chain. Both tumor types do not differ with respect to the overall usage of TCR-VDJβ genes. Productive frequencies of TCR-VDJβ genes with a prevalence of > 1% are shown in a heatmap

Supplementary methods

Data processing after whole exome and RNA sequencing

Reads were extracted from the raw exome sequencing data using CASAVA 1.8.2 (Illumina) and aligned to the human reference genome (UCSC's hg19) using the Burrows-Wheeler alignment tool, BWA 0.7.3a. The BAM files were then processed using various tools from the Picard Software Suite (v1.90) as well as tools from the Genome Analysis Toolkit (GATK, v2.5.2). Variant calling was performed using the GATK HaplotypeCaller. Matched normal control (blood) samples were used to identify somatic events. Filtering of the variants was done using the VariantQualityScoreRecalibration module from GATK and using a somatic score cut-off of > 34 and > 3 reads in both tumor and control, respectively. Somatic indels were filtered using a minimum variant frequency of 25% and > 4 reads in both tumor and control.

Reads were extracted from the raw RNA-seq data using CASAVA 1.8.2 (Illumina) and aligned to the human reference genome (UCSC's hg19) using the STAR (2.5.0c) splice aware aligner and gencode v19 transcriptome annotations. The BAM files were then processed as described above. Read counts per exon / gene were then determined by the featureCounts function of the subread package (v1.4.6-p1). The raw read counts were then normalized through the FPKM methodology.

Supplementary table 1

Patient characteristics and analyses (TIL numbers and location).

PA diagnosis	Tumor material	IDH status	Patient # (flow cytometry)	Patient # (IF)
Astrocytoma (grade II)	Biopsy	IDHmut	1	
Oligoastrocytoma (grade II)	Biopsy	IDHmut	2	
Astrocytoma (grade II)	Biopsy	IDHmut	3	
Oligodendroglioma (grade II)	Biopsy	IDHmut	4	1
Astrocytoma (grade II)	Biopsy	IDHmut	5	
Oligodendroglioma (grade II)	Biopsy	IDHmut	6	2
Oligodendroglioma (grade II)	CUSA	IDHmut	7	3
Astrocytoma (grade II)	Biopsy	IDHmut	8	
Oligodendroglioma (grade II)	CUSA	IDHmut	9	
Oligodendroglioma (grade II)	Biopsy	IDHmut	10	4
Oligodendroglioma (grade II)	Biopsy	IDHmut	11	5
Astrocytoma (grade II)	Biopsy	IDHmut	12	
Astrocytoma (grade II)	Biopsy	IDHmut		6
Oligo-astrocytoma (grade II)	Biopsy	IDHmut		7
Astrocytoma (grade II)	Biopsy	IDHmut		8
Oligo-astrocytoma (grade II)	Biopsy	IDHmut		9
Oligodendroglioma (grade II)	Biopsy	IDHmut		10
Oligo-astrocytoma (grade II)	Biopsy	IDHmut		11
Astrocytoma (grade II)	Biopsy	IDHmut		12
Astrocytoma (grade II)	Biopsy	IDHmut		13
Oligodendroglioma (grade II)	Biopsy	IDHmut		14
Oligo-astrocytoma (grade II)	Biopsy	IDHwt		15
Astrocytoma (grade II)	Biopsy	IDHmut		16
Astrocytoma (grade II)	Biopsy	IDHmut		17
Oligo-astrocytoma (grade II)	Biopsy	IDHmut		18
Oligodendroglioma (grade II)	Biopsy	IDHmut		19
Oligodendroglioma (grade II)	Biopsy	IDHmut		20
Oligodendroglioma (grade II)	Biopsy	IDHmut		21
Oligodendroglioma (grade II)	Biopsy	IDHmut		22
Oligodendroglioma (grade II)	Biopsy	IDHmut		23
Astrocytoma (grade II)	Biopsy	IDHmut		24
Oligodendroglioma (grade II)	Biopsy	IDHmut		25
Oligodendroglioma (grade II)	Biopsy	IDHmut		26
Oligodendroglioma (grade II)	Biopsy	IDHmut		27
Astrocytoma (grade II)	Biopsy	IDHmut		28

Glioblastoma (grade IV)	Biopsy	Unknown	1	
Glioblastoma (grade IV)	Biopsy	IDHmut	2	1
Glioblastoma (grade IV)	Biopsy	IDHwt	3	2
Glioblastoma (grade IV)	Biopsy	IDHwt	4	3
Glioblastoma (grade IV)	Biopsy	IDHwt	5	4
Glioblastoma (grade IV)	Biopsy	IDHwt	6	5
Glioblastoma (grade IV)	CUSA	Unknown	7	
Glioblastoma (grade IV)	CUSA	IDHmut	8	
Glioblastoma (grade IV)	Biopsy	Unknown		6
Glioblastoma (grade IV)	Biopsy	IDHwt		7
Glioblastoma (grade IV)	Biopsy	IDHwt		8
Glioblastoma (grade IV)	Biopsy	Unknown		9
Glioblastoma (grade IV)	Biopsy	IDHwt		10
Glioblastoma (grade IV)	Biopsy	IDHwt		11
Glioblastoma (grade IV)	Biopsy	Unknown		12
Glioblastoma (grade IV)	Biopsy	IDHwt		13
Glioblastoma (grade IV)	Biopsy	IDHwt		14
Glioblastoma (grade IV)	Biopsy	Unknown		15
Glioblastoma (grade IV)	Biopsy	Unknown		16
Glioblastoma (grade IV)	Biopsy	IDHwt		17
Glioblastoma (grade IV)	Biopsy	IDHwt		18
Glioblastoma (grade IV)	Biopsy	IDHwt		19
Glioblastoma (grade IV)	Biopsy	IDHwt		20
Glioblastoma (grade IV)	Biopsy	IDHwt		21
Glioblastoma (grade IV)	Biopsy	Unknown		22
Glioblastoma (grade IV)	Biopsy	IDHwt		23
Glioblastoma (grade IV)	Biopsy	IDHwt		24
Glioblastoma (grade IV)	Biopsy	IDHwt		25
Glioblastoma (grade IV)	Biopsy	Unknown		26
Glioblastoma (grade IV)	Biopsy	IDHwt		27
Glioblastoma (grade IV)	Biopsy	Unknown		28

Supplementary table 2

Patient characteristics and analyses (NGS).

Tumor #	Integrated diagnosis	Tumor material	HLA A	HLA-B	HLA-C
1	Glioblastoma (grade IV), IDH mutant	FF	A26:01, A02:01	B44:02, B56:01	C01:02, C05:01
2	Glioblastoma (grade IV), IDH wild-type	FFPE	A29:02, A02:01	B55:01, B44:02	C05:01, C03:03
3	Glioblastoma (grade IV), IDH wild-type	FFPE	A01:01, A33:01	B14:02, B08:01	C08:02, C07:01
4	Glioblastoma (grade IV), IDH wild-type	FF	A01:01, A03:01	B18:01, B07:02	C06:02, C07:02
5	Glioblastoma (grade IV), IDH wild-type	FF	A01:01, A30:01	B13:02, B08:01	C06:02, C07:01
1	Oligodendroglioma (grade II), IDH mutant and 1p/19q codeleted	FFPE	A32:01, A30:01	B13:02, B08:01	C04:01, C06:02
2	Oligodendroglioma (grade II), IDH mutant and 1p/19q codeleted	FFPE	A68:01, A03:01	B56:01, B35:01	C04:01, C01:02
3	Diffuse astrocytoma (grade II), IDH mutant	FF	A26:01, A02:01	B44:02, B15:08	C05:01, C01:02
4	Oligodendroglioma (grade II), IDH mutant and 1p/19q codeleted	FF	A01:01, A03:01	B08:01, B07:02	C07:02, C07:02
5	Oligodendroglioma (grade II), IDH mutant and 1p/19q codeleted	FF	A02:01, A02:01	B44:03, B18:01	C06:02, C07:01

Supplementary table 3

Number of predicted neo-antigens in 5 LGG and 5 HGG

Tumor #	Histology	Somatic mutations	Nonsyn. mutations in expressed genes	Potential neoantigens
1	Glioblastoma (grade IV)	62	23	2
2	Glioblastoma (grade IV)	51	20	2
3	Glioblastoma (grade IV)	75	29	0
4	Glioblastoma (grade IV)	35	17	1
5	Glioblastoma (grade IV)	74	35	6
1	Oligodendroglioma (grade II)	18	9	2
2	Oligodendroglioma (grade II)	33	14	4
3	Astrocytoma (grade II)	32	13	0
4	Oligodendroglioma (grade II)	51	19	1
5	Oligodendroglioma (grade II)	34	15	4

Chr	Start	End	Ref	Alt	Func.refgene	Gene.refgene	ExonicFunc.refGene.3bp.	NormalReads	VariantReads	VAF
chr1	3415379	3415379	C	T	exonic	MEGF6	nonsynonymous	23	20	0.47
chr1	22985938	22985938	G	A	UTR5	C1QB	.	31	29	0.48
chr1	118166138	118166138	C	T	exonic	FAM46C	synonymous	36	35	0.49
chr1	159273902	159273902	C	G	exonic	FCER1A	nonsynonymous	66	32	0.33
chr1	237947345	237947345	C	T	exonic	RYR2	synonymous	60	42	0.41
chr2	24896241	24896241	C	A	exonic	NCOA1	stopgain	52	58	0.53
chr2	43801863	43801863	C	T	exonic	THADA	synonymous	44	25	0.36
chr2	44071758	44071758	G	A	intronic	ABCG8	.	38	23	0.38
chr2	109524408	109524408	C	T	exonic	EDAR	nonsynonymous	40	29	0.42
chr2	138169325	138169325	C	T	exonic	THSD7B	unknown	46	39	0.46
chr3	156170726	156170726	G	A	splicing	KCNAB1	.	56	38	0.40
chr4	68619793	68619793	G	T	exonic	GNRHR	nonsynonymous	43	32	0.43
chr5	23526441	23526441	A	T	exonic	PRDM9	nonsynonymous	84	53	0.39
chr5	168195287	168195287	G	A	intronic	SLIT3	.	38	44	0.54
chr5	175112413	175112413	C	A	exonic	HRH2	nonsynonymous	25	17	0.40
chr6	84563159	84563159	G	C	exonic	RIPPLY2	nonsynonymous	10	5	0.33
chr6	158925041	158925041	G	A	exonic	TULP4	nonsynonymous	43	50	0.54
chr7	24911582	24911582	C	A	exonic	OSBPL3	nonsynonymous	128	99	0.44
chr7	50455009	50455009	A	T	intronic	IKZF1	.	115	55	0.32
chr8	6735328	6735328	T	G	exonic	DEFB1	nonsynonymous	121	73	0.38
chr8	41571762	41571762	G	A	exonic	ANK1	nonsynonymous	51	46	0.47
chr8	61754386	61754386	A	G	intronic	CHD7	.	57	37	0.39
chr8	88298751	88298751	T	C	intronic	CNBD1	.	37	28	0.43
chr8	146279383	146279383	C	T	intronic	C8orf33	.	36	26	0.42
chr11	124135295	124135295	G	A	exonic	OR8G5	synonymous	42	38	0.48
chr11	128781644	128781644	A	G	exonic	KCNJ5	nonsynonymous	39	34	0.47
chr12	4479511	4479511	A	T	exonic	FGF23	stoploss	51	23	0.31
chr12	6970130	6970130	G	A	exonic	USP5	nonsynonymous	60	43	0.42
chr12	10233952	10233952	C	G	exonic	CLEC1A	nonsynonymous	81	52	0.39
chr12	11461570	11461570	G	T	exonic	PRB4	nonsynonymous	6	9	0.60
chr12	119631579	119631579	C	A	exonic	HSPB8	synonymous	56	29	0.34
chr14	22038342	22038342	G	C	exonic	OR10G3	nonsynonymous	137	71	0.34
chr14	102504830	102504830	G	A	exonic	DYNC1H1	nonsynonymous	18	96	0.84
chr15	43678543	43678543	G	A	ncRNA_intror	RNU6-28P	.	12	18	0.60
chr15	63419597	63419597	C	T	exonic	LACTB	nonsynonymous	29	23	0.44
chr16	321398	321398	G	A	exonic	RGS11	nonsynonymous	48	42	0.47
chr17	36491003	36491003	C	T	exonic	GPR179	nonsynonymous	12	13	0.52
chr17	48655820	48655820	C	G	exonic	CACNA1G	nonsynonymous	43	63	0.59
chr17	76570842	76570842	C	T	exonic	DNAH17	nonsynonymous	67	48	0.42
chr18	77171379	77171379	C	T	exonic	NFATC1	synonymous	29	19	0.40
chr19	7809880	7809880	C	T	exonic	CD209	nonsynonymous	98	70	0.42
chr19	10737010	10737010	G	A	intronic	SLC44A2	.	72	33	0.31
chr19	16611916	16611916	A	G	exonic	C19orf44	nonsynonymous	54	50	0.48
chr19	22363083	22363083	C	T	exonic	ZNF676	nonsynonymous	24	18	0.43
chr19	22846668	22846668	T	A	exonic	ZNF492	nonsynonymous	66	45	0.41
chr20	44669236	44669236	C	T	exonic	SLC12A5	synonymous	52	41	0.44
chr21	38081587	38081587	G	A	intronic	SIM2	.	16	8	0.33
chrX	35820608	35820608	T	G	exonic	MAGEB16	nonsynonymous	8	43	0.84
chrX	53586382	53586382	G	A	exonic	HUWE1	synonymous	3	32	0.91
chr1	16782343	16782343	A	G	exonic	NECAP2	nonsynonymous	43	30	0.41
chr1	21807521	21807521	A	T	intronic	NBPF3	.	26	9	0.26
chr1	25344442	25344442	C	T	intergenic	RUNX3,SYF2	.	49	26	0.35
chr1	182443253	182443253	C	G	exonic	RGSL1	nonsynonymous	47	31	0.40
chr1	196796036	196796036	C	T	exonic	CFHR1	stopgain	105	47	0.31
chr1	205632009	205632009	C	T	exonic	SLC45A3	nonsynonymous	40	27	0.40
chr2	106509636	106509636	C	T	UTR3	NCK2	.	57	45	0.44
chr2	179464326	179464326	T	G	exonic	TTN	nonsynonymous	37	30	0.45
chr2	219146894	219146894	G	T	UTR5	TMBIM1	.	7	3	0.30
chr3	10370599	10370599	T	C	exonic	ATP2B2	nonsynonymous	78	47	0.38
chr3	193051604	193051604	C	T	exonic	ATP13A5	nonsynonymous	72	40	0.36
chr4	20550097	20550097	T	C	intronic	SLIT2	.	46	30	0.39

chr4	71063840	71063840	A	T	exonic	ODAM	nonsynonymous	43	27	0.39
chr4	71064384	71064384	T	A	intronic	ODAM	.	40	34	0.46
chr4	79334262	79334262	G	A	intronic	FRAS1	.	72	36	0.33
chr5	40843707	40843707	A	G	exonic	CARD6	nonsynonymous	74	37	0.33
chr5	49695043	49695043	A	G	UTR3	EMB	.	34	22	0.39
chr5	112328444	112328444	C	T	exonic	DCP2	nonsynonymous	76	43	0.36
chr5	176918778	176918778	G	A	intronic	PDLIM7	.	20	16	0.44
chr6	25726546	25726546	C	T	exonic	HIST1H2AA	synonymous	58	35	0.38
chr6	83075081	83075081	G	A	exonic	TPBG	nonsynonymous	17	8	0.32
chr6	159667972	159667972	C	T	exonic	FNDC1	nonsynonymous	61	52	0.46
chr6	168708321	168708321	C	A	exonic	DACT2	nonsynonymous	22	17	0.44
chr7	21639605	21639605	G	A	exonic	DNAH11	synonymous	92	41	0.31
chr7	34917702	34917702	G	A	exonic	NPSR1	nonsynonymous	57	28	0.33
chr7	71175875	71175875	G	T	exonic	WBSCR17	nonsynonymous	70	25	0.26
chr7	94039107	94039107	G	A	exonic	COL1A2	nonsynonymous	171	73	0.30
chr7	138391393	138391393	G	C	exonic	ATP6V0A4	nonsynonymous	96	44	0.31
chr8	1497384	1497384	C	T	exonic	DLGAP2	synonymous	35	31	0.47
chr8	12892759	12892759	G	T	intergenic	KIAA1456,D	.	65	68	0.51
chr8	36694400	36694400	A	T	exonic	KCNU1	nonsynonymous	91	54	0.37
chr8	110455153	110455153	G	A	intronic	PKHD1L1	.	26	21	0.45
chr8	120255673	120255673	C	T	exonic	MAL2	nonsynonymous	98	50	0.34
chr9	6014444	6014444	C	T	exonic	RANBP6	nonsynonymous	35	31	0.47
chr9	40774086	40774086	C	A	exonic	ZNF658	nonsynonymous	64	44	0.41
chr9	116345872	116345872	A	G	exonic	RGS3	nonsynonymous	72	26	0.27
chr9	124525882	124525882	G	A	exonic	DAB2IP	synonymous	29	41	0.59
chr10	45935934	45935934	C	T	exonic	ALOX5	synonymous	27	58	0.68
chr11	14865403	14865403	T	C	exonic	PDE3B	nonsynonymous	62	33	0.35
chr12	18499703	18499703	G	A	exonic	PIK3C2G	nonsynonymous	38	16	0.30
chr12	71002958	71002958	C	A	exonic	PTPRB	synonymous	71	39	0.35
chr12	81043315	81043315	C	T	intronic	PTPRQ	.	40	22	0.35
chr12	102147187	102147187	G	A	exonic	GNPTAB	stopgain	70	37	0.35
chr12	117723112	117723112	G	A	exonic	NOS1	nonsynonymous	63	55	0.47
chr14	35253077	35253077	T	C	exonic	BAZ1A	nonsynonymous	60	37	0.38
chr14	106311716	106311716	C	T	intergenic	ELK2AP,KIA/	.	26	21	0.45
chr15	43021977	43021977	G	A	intronic	CDAN1	.	34	23	0.40
chr15	79382633	79382633	C	T	exonic	RASGRF1	nonsynonymous	22	15	0.41
chr15	91825028	91825028	G	A	exonic	SV2B	nonsynonymous	54	27	0.33
chr16	1272219	1272219	G	A	exonic	TPSG1	nonsynonymous	13	13	0.50
chr16	2024443	2024443	G	A	intronic	TBL3	.	34	32	0.48
chr16	3299706	3299706	G	A	exonic	MEFV	nonsynonymous	65	26	0.29
chr16	20807832	20807832	G	T	UTR3	ERI2	.	58	25	0.30
chr17	3833651	3833651	G	A	exonic	ATP2A3	synonymous	85	54	0.39
chr17	10346874	10346874	A	G	intronic	MYH4	.	40	16	0.29
chr17	37886722	37886722	G	A	exonic	MIEN1	synonymous	40	21	0.34
chr17	40706946	40706946	C	T	exonic	HSD17B1	nonsynonymous	3	9	0.75
chr17	61842182	61842182	T	C	exonic	CCDC47	nonsynonymous	40	31	0.44
chr17	62080169	62080169	G	A	exonic	ICAM2	nonsynonymous	48	29	0.38
chr18	47527697	47527697	G	A	exonic	MYO5B	synonymous	87	57	0.40
chr18	61765114	61765114	C	T	ncRNA_exoni	LINC00305	.	49	36	0.42
chr18	63547805	63547805	G	A	exonic	CDH7	nonsynonymous	43	36	0.46
chr19	6772883	6772883	G	A	exonic	VAV1	nonsynonymous	59	29	0.33
chr19	45026779	45026779	G	A	exonic	CEACAM20	nonsynonymous	82	40	0.33
chr19	48003875	48003875	C	T	ncRNA_intror	NAPA-AS1	.	65	31	0.32
chr20	33554057	33554057	G	A	intronic	MYH7B	.	57	30	0.34
chr20	62843515	62843515	A	G	intronic	MYT1	.	23	40	0.63
chr21	40981600	40981600	G	A	ncRNA_intror	C21orf88	.	49	19	0.28
chr22	42126566	42126566	G	A	exonic	May-01	nonsynonymous	43	40	0.48
chr22	51012784	51012784	C	A	exonic	CPT1B	nonsynonymous	46	31	0.40
chrX	142795246	142795246	A	G	exonic	SPANXN2	synonymous	8	36	0.82
chr2	209113112	209113112	C	T	exonic	IDH1	nonsynonymous	27	26	0.49
chr2	211421462	211421462	C	T	exonic	CPS1	nonsynonymous	59	43	0.42
chr3	119188681	119188681	G	A	exonic	POGLUT1	nonsynonymous	92	50	0.35
chr4	184256375	184256375	C	T	intergenic	CLDN24,CDI	.	4	7	0.64

chr7	43921215	43921215	C	G	intronic	URGCP,URG	.	54	45	0.45
chr7	82763762	82763762	G	C	exonic	PCLO	nonsynonymous	45	31	0.41
chr9	96082594	96082594	C	T	intronic	C9orf129,W	.	67	35	0.34
chr11	2905239	2905239	G	A	exonic	CDKN1C	nonsynonymous	16	11	0.41
chr11	107675382	107675382	T	A	intronic	SLC35F2	.	8	5	0.38
chr19	36340205	36340205	C	A	exonic	NPHS1	nonsynonymous	18	22	0.55
chr19	44417645	44417645	C	G	exonic	ZNF45	nonsynonymous	17	17	0.50
chrX	70357220	70357220	G	A	exonic	MED12	nonsynonymous	55	33	0.38
chr1	205897095	205897095	C	T	exonic	SLC26A9	nonsynonymous	61	63	0.51
chr1	236577649	236577649	T	A	intronic	EDARADD	.	45	36	0.44
chr2	102503629	102503629	G	T	exonic	MAP4K4	nonsynonymous	66	45	0.41
chr2	209113112	209113112	C	T	exonic	IDH1	nonsynonymous	28	25	0.47
chr3	52858433	52858433	G	A	exonic	ITIH4	nonsynonymous	48	50	0.51
chr3	56680835	56680835	C	T	exonic	FAM208A	nonsynonymous	30	18	0.38
chr3	78667105	78667105	T	C	exonic	ROBO1	nonsynonymous	50	30	0.38
chr3	184041717	184041717	G	C	exonic	EIF4G1	nonsynonymous	54	34	0.39
chr4	123329206	123329206	A	G	intronic	ADAD1	.	11	18	0.62
chr5	139917128	139917128	C	T	exonic	ANKHD1,AN	synonymous	100	57	0.36
chr6	43410860	43410860	G	A	exonic	ABCC10	synonymous	33	16	0.33
chr6	166921761	166921761	G	A	exonic	RPS6KA2	nonsynonymous	71	65	0.48
chr8	52321396	52321396	C	T	exonic	PXDNL	nonsynonymous	33	23	0.41
chr8	101059734	101059734	G	T	exonic	RGS22	nonsynonymous	71	46	0.39
chr8	144773245	144773245	G	T	exonic	ZNF707	nonsynonymous	73	72	0.50
chr9	34490446	34490446	T	C	exonic	DNAI1	nonsynonymous	93	81	0.47
chr9	116972011	116972011	A	T	intronic	COL27A1	.	62	42	0.40
chr9	131010180	131010180	A	G	intronic	DNM1	.	57	32	0.36
chr12	116424105	116424105	T	C	exonic	MED13L	nonsynonymous	63	45	0.42
chr13	19751365	19751365	G	A	exonic	TUBA3C	nonsynonymous	129	69	0.35
chr13	36049784	36049784	A	G	exonic	MAB21L1	synonymous	37	40	0.52
chr16	81953233	81953233	C	T	exonic	PLCG2	synonymous	95	62	0.39
chr17	42461422	42461422	C	T	intronic	ITGA2B	.	40	30	0.43
chr19	6773006	6773006	G	A	exonic	VAV1	nonsynonymous	30	17	0.36
chr19	8503309	8503309	G	A	exonic	2-Mar	stopgain	61	78	0.56
chr19	18991094	18991094	G	A	exonic	CERS1	synonymous	43	25	0.37
chr19	42791718	42791718	C	T	exonic	CIC	nonsynonymous	4	30	0.88
chr22	19965472	19965472	C	T	intronic	ARVCF	.	35	24	0.41
chr1	17413063	17413063	A	G	exonic	PADI2	nonsynonymous	74	63	0.46
chr1	117142742	117142742	G	T	exonic	IGSF3	nonsynonymous	28	33	0.54
chr1	183094548	183094548	G	A	exonic	LAMC1	synonymous	53	31	0.37
chr1	202531976	202531976	C	T	exonic	PPP1R12B	nonsynonymous	115	100	0.47
chr1	212002487	212002487	C	A	exonic	LPGAT1	nonsynonymous	59	62	0.51
chr2	27659642	27659642	T	A	exonic	NRBP1	nonsynonymous	34	31	0.48
chr2	135883759	135883759	A	G	exonic	RAB3GAP1	nonsynonymous	79	57	0.42
chr2	169939968	169939968	T	C	exonic	DHRS9	nonsynonymous	61	45	0.42
chr2	189962037	189962037	C	T	exonic	COL5A2	nonsynonymous	90	74	0.45
chr2	209113112	209113112	C	T	exonic	IDH1	nonsynonymous	34	33	0.49
chr3	39107385	39107385	G	C	intronic	WDR48	.	19	68	0.78
chr3	75788130	75788130	C	T	exonic	ZNF717	nonsynonymous	1	5	0.83
chr3	75788137	75788137	C	T	exonic	ZNF717	nonsynonymous	1	5	0.83
chr3	75788152	75788152	T	C	exonic	ZNF717	nonsynonymous	1	5	0.83
chr3	75788192	75788192	T	C	exonic	ZNF717	synonymous	3	3	0.50
chr3	183906140	183906140	C	T	exonic	ABCF3	stopgain	37	32	0.46
chr4	110790918	110790918	A	T	exonic	LRIT3	nonsynonymous	55	60	0.52
chr4	156632085	156632085	C	T	exonic	GUCY1A3	synonymous	71	54	0.43
chr4	186366609	186366609	A	G	UTR3	CCDC110	.	58	60	0.51
chr5	42762544	42762544	G	C	splicing	CCDC152	.	81	65	0.45
chr6	36949423	36949423	G	A	exonic	MTCH1	nonsynonymous	44	61	0.58
chr6	53140060	53140060	C	G	splicing	ELOVL5	.	96	83	0.46
chr6	62407037	62407037	T	C	intronic	KHDRBS2	.	35	25	0.42
chr6	62407045	62407045	G	T	intronic	KHDRBS2	.	37	26	0.41
chr6	129763435	129763435	A	T	intronic	LAMA2	.	11	53	0.83
chr7	103777209	103777209	A	G	intronic	ORC5	.	71	55	0.44
chr7	138424275	138424275	A	C	intronic	ATP6V0A4	.	102	156	0.60

chr8	37695246	37695246	T	C	intronic	GPR124	.	15	24	0.62
chr8	119391817	119391817	G	A	exonic	SAMD12	synonymous	37	34	0.48
chr9	130898230	130898230	C	T	intergenic	PTGES2-AS1	.	36	33	0.48
chr11	418774	418774	G	A	exonic	ANO9	synonymous	56	49	0.47
chr11	64087199	64087199	C	T	intronic	PRDX5	.	56	42	0.43
chr11	66330521	66330521	G	A	exonic	ACTN3	unknown	52	34	0.40
chr11	119513809	119513809	A	G	intronic	PVRL1	.	57	55	0.49
chr12	49720397	49720397	C	T	intronic	TROAP	.	25	26	0.51
chr12	58124268	58124268	C	T	intronic	AGAP2	.	11	51	0.82
chr14	88414252	88414252	A	G	intronic	GALC	.	26	14	0.35
chr14	93761087	93761087	C	T	exonic	BTBD7	stopgain	6	33	0.85
chr14	93761088	93761088	C	A	exonic	BTBD7	nonsynonymous	6	33	0.85
chr15	41961236	41961236	T	C	exonic	MGA	synonymous	110	39	0.26
chr15	64275832	64275832	C	T	exonic	DAPK2	nonsynonymous	43	26	0.38
chr16	822738	822738	C	T	intergenic	MIR662,RPL	.	12	12	0.50
chr16	1257298	1257298	C	T	exonic	CACNA1H	synonymous	43	38	0.47
chr16	22335668	22335668	C	T	intronic	POLR3E	.	139	53	0.28
chr16	48141317	48141317	G	A	exonic	ABCC12	synonymous	49	49	0.50
chr17	7577547	7577547	C	G	exonic	TP53	nonsynonymous	3	81	0.96
chr17	16676841	16676841	T	C	exonic	CCDC144A	stoploss	72	41	0.36
chr17	19646604	19646604	C	T	exonic	ALDH3A1	nonsynonymous	45	19	0.30
chr17	37901951	37901951	G	A	intronic	GRB7	.	22	17	0.44
chr17	54985907	54985907	T	C	exonic	TRIM25	synonymous	23	18	0.44
chr19	6454575	6454575	T	C	exonic	SLC25A23	nonsynonymous	31	18	0.37
chr19	47861352	47861352	C	T	exonic	DHX34	nonsynonymous	4	18	0.82
chr19	53057759	53057759	T	C	exonic	ZNF808	synonymous	27	22	0.45
chr22	24581758	24581758	A	T	exonic	SUSD2	synonymous	43	15	0.26
chr22	37870732	37870732	G	T	intronic	MFNG	.	39	42	0.52
chr22	38995804	38995804	C	A	exonic	FAM227A	synonymous	63	40	0.39
chr22	38995805	38995805	A	C	exonic	FAM227A	nonsynonymous	64	40	0.38
chrX	48838236	48838236	A	G	exonic	GRIPAP1	nonsynonymous	3	36	0.92
chr1	8385928	8385928	G	A	exonic	SLC45A1	nonsynonymous	56	34	0.38
chr1	24454733	24454733	C	T	exonic	IL22RA1	nonsynonymous	59	54	0.48
chr1	57202850	57202850	A	T	intronic	C1orf168	.	37	34	0.48
chr1	240371121	240371121	T	A	exonic	FMN2	synonymous	2	2	0.50
chr2	677352	677352	G	T	UTR5	TMEM18	.	57	29	0.34
chr2	207509116	207509116	C	G	exonic	LOC200726	synonymous	39	23	0.37
chr4	8621188	8621188	C	T	exonic	CPZ	synonymous	41	25	0.38
chr4	76817425	76817425	T	C	exonic	PPEF2	synonymous	60	38	0.39
chr5	10254820	10254820	T	A	exonic	CCT5	nonsynonymous	45	80	0.64
chr6	26409748	26409748	G	A	intronic	BTN3A1	.	31	29	0.48
chr7	81340799	81340799	T	A	exonic	HGF	nonsynonymous	28	14	0.33
chr8	113418904	113418904	G	T	exonic	CSMD3	nonsynonymous	99	58	0.37
chr9	113198713	113198713	C	A	exonic	SVEP1	nonsynonymous	74	40	0.35
chr11	621606	621606	C	A	exonic	CDHR5	nonsynonymous	14	19	0.58
chr12	9313635	9313635	C	G	exonic	PZP	nonsynonymous	43	54	0.56
chr12	101750778	101750778	C	T	exonic	UTP20	nonsynonymous	30	29	0.49
chr13	25914232	25914232	T	A	exonic	NUPL1	stopgain	28	46	0.62
chr13	48934155	48934155	G	T	exonic	RB1	stopgain	24	25	0.51
chr14	20612024	20612024	C	A	exonic	OR4N5	nonsynonymous	73	45	0.38
chr14	24806093	24806093	G	C	exonic	RIPK3	nonsynonymous	61	38	0.38
chr15	84488672	84488672	C	T	exonic	ADAMTSL3	nonsynonymous	39	30	0.43
chr17	7578212	7578212	G	A	exonic	TP53	stopgain	50	98	0.66
chr17	29654694	29654694	G	T	exonic	NF1	stopgain	38	111	0.74
chr17	38928015	38928015	G	A	exonic	KRT26	synonymous	13	39	0.75
chr19	30934835	30934835	C	T	exonic	ZNF536	synonymous	18	13	0.42
chr19	39264116	39264116	G	T	intronic	LGALS7	.	8	5	0.38
chr19	54802546	54802546	C	T	exonic	LILRA3	nonsynonymous	21	17	0.45
chr22	44324694	44324694	G	A	intronic	PNPLA3	.	51	40	0.44
chrX	76855002	76855002	C	A	exonic	ATRX	nonsynonymous	18	40	0.69
chrX	119037243	119037243	C	T	exonic	AKAP14	synonymous	28	59	0.68
chr1	6646083	6646083	C	T	exonic	ZBTB48	nonsynonymous	108	50	0.32
chr1	40034556	40034556	C	T	exonic	PABPC4	nonsynonymous	48	20	0.29

chr1	169757984	169757984	A	T	intergenic	SELE,METTL	.	57	33	0.37
chr2	96799737	96799737	C	T	exonic	ASTL	nonsynonymous	41	18	0.31
chr2	103318927	103318927	T	C	exonic	SLC9A2	nonsynonymous	125	43	0.26
chr2	119699964	119699964	G	A	exonic	MARCO	nonsynonymous	107	40	0.27
chr3	71804123	71804123	T	A	exonic	GPR27	nonsynonymous	18	11	0.38
chr3	129233244	129233244	C	T	exonic	IFT122	synonymous	22	15	0.41
chr3	178916600	178916600	A	G	UTR5	PIK3CA	.	47	23	0.33
chr3	186501404	186501404	C	G	exonic	EIF4A2	nonsynonymous	80	47	0.37
chr4	15341859	15341859	T	A	intronic	C1QTNF7	.	64	53	0.45
chr5	36207263	36207263	G	A	intronic	NADK2	.	71	63	0.47
chr5	39074433	39074433	C	G	exonic	RICTOR	nonsynonymous	25	24	0.49
chr5	45303809	45303809	G	A	exonic	HCN1	stopgain	114	44	0.28
chr5	79373951	79373951	C	T	exonic	THBS4	synonymous	64	24	0.27
chr6	152665277	152665277	A	G	exonic	SYNE1	nonsynonymous	59	21	0.26
chr7	47897311	47897311	G	A	exonic	PKD1L1	synonymous	61	21	0.26
chr7	103236889	103236889	A	G	intronic	RELN	.	61	24	0.28
chr7	142423715	142423715	C	A	intergenic	MTRNR2L6,I	.	17	12	0.41
chr8	41522562	41522562	G	A	UTR5	ANK1	.	14	12	0.46
chr8	113395804	113395804	C	A	exonic	CSMD3	nonsynonymous	53	25	0.32
chr8	120435220	120435220	A	G	exonic	NOV	nonsynonymous	28	16	0.36
chr9	339016	339016	G	A	exonic	DOCK8	nonsynonymous	57	28	0.33
chr9	19049675	19049675	G	A	exonic	RRAGA	nonsynonymous	45	16	0.26
chr9	138657033	138657033	C	T	exonic	KCNT1	nonsynonymous	46	25	0.35
chr10	49951409	49951409	C	T	exonic	WDFY4	nonsynonymous	18	19	0.51
chr10	73571748	73571748	A	T	exonic	CDH23	nonsynonymous	17	32	0.65
chr10	89692905	89692905	G	A	exonic	PTEN	nonsynonymous	34	57	0.63
chr10	118028966	118028966	C	T	intronic	GFRA1	.	42	73	0.63
chr11	4928591	4928591	T	A	upstream	OR51A7	.	21	13	0.38
chr11	6432276	6432276	T	A	exonic	APBB1	nonsynonymous	29	16	0.36
chr11	61897619	61897619	C	T	exonic	INCENP	nonsynonymous	29	19	0.40
chr11	65622776	65622776	C	T	UTR3	CFL1	.	12	14	0.54
chr12	665816	665816	G	A	exonic	B4GALNT3	nonsynonymous	49	21	0.30
chr12	47170743	47170743	G	A	exonic	SLC38A4	nonsynonymous	69	60	0.47
chr12	48189778	48189778	G	A	exonic	HDAC7	nonsynonymous	7	8	0.53
chr12	85255682	85255682	C	T	exonic	SLC6A15	nonsynonymous	110	42	0.28
chr12	119563207	119563207	G	T	exonic	SRRM4	nonsynonymous	37	25	0.40
chr12	122614568	122614568	C	T	exonic	MLXIP	synonymous	52	19	0.27
chr12	129180447	129180447	C	T	exonic	TMEM132C	synonymous	27	17	0.39
chr12	132498322	132498322	G	A	exonic	EP400	nonsynonymous	123	57	0.32
chr13	42869921	42869921	G	C	intronic	AKAP11	.	46	23	0.33
chr14	19685473	19685473	C	T	ncRNA_intror	DUXAP10	.	4	4	0.50
chr14	22554913	22554913	A	T	intergenic	OR4E2,DAD	.	86	34	0.28
chr16	31373161	31373161	G	A	exonic	ITGAX	synonymous	34	22	0.39
chr16	53726197	53726197	G	T	exonic	RPGRIP1L	synonymous	48	24	0.33
chr17	1630295	1630295	C	T	exonic	WDR81	nonsynonymous	23	10	0.30
chr17	7577120	7577120	C	T	exonic	TP53	nonsynonymous	74	45	0.38
chr18	13885234	13885234	C	T	exonic	MC2R	nonsynonymous	34	18	0.35
chr18	28916567	28916567	C	T	exonic	DSG1	nonsynonymous	54	26	0.33
chr18	77063666	77063666	G	A	exonic	ATP9B	nonsynonymous	45	16	0.26
chr19	8808028	8808028	C	T	exonic	ACTL9	nonsynonymous	24	10	0.29
chr19	16682362	16682362	G	T	exonic	SLC35E1	synonymous	62	26	0.30
chr19	41135433	41135433	C	T	exonic	LTBP4	unknown	13	9	0.41
chr19	54206018	54206018	G	A	ncRNA_exoni	MIR518B	.	85	39	0.31
chr20	1532602	1532602	G	A	exonic	SIRPD	synonymous	79	30	0.28
chr21	19737470	19737470	G	T	exonic	TMPRSS15	nonsynonymous	137	71	0.34
chr21	19737492	19737492	A	T	exonic	TMPRSS15	synonymous	151	79	0.34
chr22	41513220	41513220	G	A	exonic	EP300	nonsynonymous	64	27	0.30
chr22	43608539	43608539	C	T	exonic	SCUBE1	nonsynonymous	62	22	0.26
chrX	13645158	13645158	C	T	exonic	EGFL6	synonymous	70	29	0.29
chrX	38031210	38031210	T	A	exonic	SRPX	synonymous	74	25	0.25
chrX	39817722	39817722	G	T	intergenic	LINC01282,I	.	50	27	0.35
chrX	48780887	48780887	C	A	intronic	OTUD5	.	34	15	0.31
chrX	49105291	49105291	A	T	exonic	CCDC22	nonsynonymous	54	22	0.29

chrX	55020401	55020401	G	A	exonic	PFKFB1	stopgain	52	23	0.31
chrX	74644493	74644493	T	C	exonic	ZDHHC15	nonsynonymous	109	49	0.31
chrX	77912719	77912719	G	A	exonic	ZCCHC5	nonsynonymous	70	31	0.31
chrX	78426850	78426850	C	T	exonic	GPR174	stopgain	31	24	0.44
chrX	89177180	89177180	C	A	exonic	TGIF2LX	synonymous	138	61	0.31
chrX	153668311	153668311	C	T	exonic	GDI1	nonsynonymous	66	33	0.33
chr1	43774787	43774787	C	T	exonic	TIE1	synonymous	38	19	0.33
chr1	200014659	200014659	G	A	exonic	NR5A2	nonsynonymous	100	71	0.42
chr1	216390842	216390842	T	A	exonic	USH2A	nonsynonymous	60	43	0.42
chr1	243675656	243675656	G	C	exonic	AKT3	nonsynonymous	76	32	0.30
chr2	158142611	158142611	T	C	exonic	GALNT5	nonsynonymous	105	92	0.47
chr2	209113112	209113112	C	T	exonic	IDH1	nonsynonymous	33	29	0.47
chr2	226516263	226516263	G	A	exonic	NYAP2	synonymous	55	37	0.40
chr3	47632323	47632323	C	G	exonic	SMARCC1	nonsynonymous	21	17	0.45
chr3	119153564	119153564	A	C	intronic	TMEM39A	.	33	25	0.43
chr4	71024167	71024167	T	A	exonic	PRR27	synonymous	26	19	0.42
chr4	74347573	74347573	G	A	exonic	AFM	synonymous	61	47	0.44
chr5	60821717	60821717	T	G	exonic	ZSWIM6	nonsynonymous	60	38	0.39
chr7	56149845	56149845	G	A	intronic	PHKG1	.	44	31	0.41
chr7	141536328	141536328	G	A	exonic	PRSS37	nonsynonymous	37	23	0.38
chr8	143846518	143846518	G	A	exonic	LYNX1	synonymous	23	25	0.52
chr9	135116375	135116375	C	T	exonic	NTNG2	nonsynonymous	36	15	0.29
chr12	9085274	9085274	C	T	exonic	PHC1	synonymous	45	42	0.48
chr12	9085855	9085855	C	T	exonic	PHC1	nonsynonymous	8	12	0.60
chr14	24030714	24030714	T	C	ncRNA_exoni	LOC102724	.	24	18	0.43
chr15	43678543	43678543	G	A	ncRNA_intror	RNU6-28P	.	8	11	0.58
chr16	88802830	88802830	A	G	ncRNA_intror	LOC100289	.	17	13	0.43
chr17	7577120	7577120	C	T	exonic	TP53	nonsynonymous	68	47	0.41
chr18	47511088	47511088	C	T	exonic	MYO5B	nonsynonymous	40	43	0.52
chrX	76909629	76909629	G	A	exonic	ATRX	stopgain	32	49	0.60
chrX	131206259	131206259	T	C	intronic	MST4	.	90	40	0.31
chrX	137790950	137790950	G	T	intronic	FGF13	.	40	24	0.38
chrX	153048648	153048648	T	C	intronic	SRPK3	.	7	5	0.42
chrX	154091409	154091409	A	G	exonic	F8	nonsynonymous	78	34	0.30
chr1	63788861	63788861	C	T	exonic	FOXD3	synonymous	5	18	0.78
chr1	151210662	151210662	G	A	exonic	PIP5K1A	nonsynonymous	75	63	0.46
chr1	186863234	186863234	C	T	exonic	PLA2G4A	nonsynonymous	81	58	0.42
chr1	248737157	248737157	T	C	exonic	OR2T34	nonsynonymous	8	9	0.53
chr2	27657416	27657416	A	G	exonic	NRBP1	nonsynonymous	147	109	0.43
chr2	209113112	209113112	C	T	exonic	IDH1	nonsynonymous	30	27	0.47
chr2	225657770	225657770	C	T	exonic	DOCK10	synonymous	48	20	0.29
chr2	228758631	228758631	C	T	exonic	DAW1	synonymous	47	40	0.46
chr3	51863533	51863533	C	T	intronic	IQCF3	.	50	58	0.54
chr3	195027312	195027312	C	T	exonic	ACAP2	synonymous	45	39	0.46
chr5	1293993	1293993	G	A	exonic	TERT	synonymous	20	21	0.51
chr5	156752682	156752682	G	A	intronic	CYFIP2	.	40	35	0.47
chr6	109697888	109697888	T	A	intronic	CD164	.	25	18	0.42
chr6	109935634	109935634	A	G	exonic	AK9	synonymous	42	23	0.35
chr6	165703559	165703559	G	A	intronic	C6orf118	.	38	20	0.34
chr7	44247054	44247054	A	T	exonic	YKT6	nonsynonymous	72	49	0.40
chr7	101840264	101840264	C	T	exonic	CUX1	stopgain	67	57	0.46
chr7	116435736	116435736	C	A	exonic	MET	nonsynonymous	36	30	0.45
chr8	24167761	24167761	C	T	intronic	ADAM28	.	75	37	0.33
chr8	33451068	33451068	G	A	exonic	DUSP26	nonsynonymous	28	32	0.53
chr8	55538727	55538727	T	C	exonic	RP1	nonsynonymous	38	37	0.49
chr9	94987390	94987390	A	C	intronic	IARS	.	60	49	0.45
chr10	17126369	17126369	G	A	exonic	CUBN	synonymous	30	39	0.57
chr11	120097551	120097551	G	A	exonic	OAF	synonymous	42	40	0.49
chr12	49724339	49724339	C	T	exonic	TROAP	nonsynonymous	46	35	0.43
chr12	106704930	106704930	G	A	exonic	TCP11L2	nonsynonymous	39	29	0.43
chr12	129178566	129178566	G	T	exonic	TMEM132C	nonsynonymous	35	21	0.38
chr13	20763211	20763211	G	A	exonic	GJB2	synonymous	43	29	0.40
chr13	42891728	42891728	C	G	exonic	AKAP11	nonsynonymous	79	60	0.43

chr13	79946072	79946072	A	T	intronic	RBM26	.	61	27	0.31
chr15	28096491	28096491	G	A	intronic	OCA2	.	39	19	0.33
chr16	2147410	2147410	G	A	exonic	PKD1	nonsynonymous	34	27	0.44
chr16	4718338	4718338	G	A	intronic	MGRN1	.	61	51	0.46
chr16	65025740	65025740	G	A	exonic	CDH11	nonsynonymous	52	63	0.55
chr16	68056074	68056074	G	A	exonic	DDX28	synonymous	76	30	0.28
chr17	6663925	6663925	G	A	intronic	XAF1	.	28	22	0.44
chr18	32443889	32443889	G	C	intronic	DTNA	.	34	31	0.48
chr19	12738906	12738906	A	G	exonic	ZNF791	nonsynonymous	52	36	0.41
chr20	36386029	36386029	G	A	exonic	CTNNBL1	nonsynonymous	71	60	0.46
chr20	47244458	47244458	G	A	exonic	PREX1	nonsynonymous	17	13	0.43
chr22	50585626	50585626	A	G	intronic	MOV10L1	.	22	15	0.41
chrX	49069207	49069207	G	A	exonic	CACNA1F	stopgain	62	69	0.53
chrX	122755239	122755239	C	T	exonic	THOC2	nonsynonymous	59	47	0.44
chrX	132160156	132160156	C	T	exonic	USP26	nonsynonymous	17	11	0.39
chrX	132887946	132887946	G	A	exonic	GPC3	stopgain	40	33	0.45
chrX	149896277	149896277	G	A	intronic	MTMR1	.	97	77	0.44
chrX	152613332	152613332	C	T	UTR3	ZNF275	.	11	9	0.45
chr1	78425950	78425950	T	C	splicing	FUBP1	.	35	53	0.60
chr1	159807771	159807771	C	T	exonic	C1orf204	synonymous	68	44	0.39
chr2	84660245	84660245	C	T	intronic	SUCLG1	.	102	81	0.44
chr2	135099140	135099140	A	G	intronic	MGAT5	.	43	22	0.34
chr2	152737392	152737392	G	A	exonic	CACNB4	synonymous	50	31	0.38
chr2	172411141	172411141	C	T	exonic	CYBRD1	nonsynonymous	46	31	0.40
chr2	179604824	179604824	G	A	exonic	TTN	nonsynonymous	36	15	0.29
chr2	209113112	209113112	C	T	exonic	IDH1	nonsynonymous	32	29	0.48
chr3	9515139	9515139	C	G	exonic	SETD5	nonsynonymous	51	27	0.35
chr3	178952085	178952085	A	G	exonic	PIK3CA	nonsynonymous	58	41	0.41
chr4	48027330	48027330	A	G	exonic	NIPAL1	nonsynonymous	32	19	0.37
chr4	83857450	83857450	T	C	exonic	LIN54	synonymous	64	27	0.30
chr4	148800431	148800431	G	C	exonic	ARHGAP10	nonsynonymous	118	73	0.38
chr4	162508721	162508721	C	A	exonic	FSTL5	stopgain	78	53	0.40
chr5	175959129	175959129	G	A	exonic	RNF44	nonsynonymous	12	6	0.33
chr6	35289138	35289138	C	T	exonic	DEF6	nonsynonymous	26	26	0.50
chr6	46638839	46638839	A	G	intronic	SLC25A27	.	100	49	0.33
chr7	100778812	100778812	C	T	exonic	SERPINE1	synonymous	58	23	0.28
chr7	135263489	135263489	G	C	intronic	NUP205	.	50	25	0.33
chr9	22451673	22451673	C	T	exonic	DMRTA1	synonymous	75	45	0.38
chr9	79465456	79465456	C	A	exonic	PRUNE2	synonymous	93	57	0.38
chr11	62295357	62295357	G	T	exonic	AHNAK	nonsynonymous	102	72	0.41
chr12	70206565	70206565	G	A	exonic	RAB3IP	nonsynonymous	68	38	0.36
chr17	4402683	4402683	T	G	exonic	SPNS2	nonsynonymous	6	7	0.54
chr17	37902069	37902069	G	A	intronic	GRB7	.	27	15	0.36
chr19	2222027	2222027	G	A	exonic	DOT1L	synonymous	23	18	0.44
chr19	2223277	2223277	C	T	intronic	DOT1L	.	26	19	0.42
chrX	135092619	135092619	G	A	exonic	SLC9A6	synonymous	6	30	0.83
chrY	7194066	7194066	A	G	ncRNA_exoni	PRKY	.	13	30	0.70
chr1	153233488	153233488	-	GGCGGT	exonic	LOR	nonframeshift	15	8	0.35
chr20	48184682	48184682	-	CGGGGC	UTR5	PTGIS	.	0	8	0.89
chr3	27763427	27763427	-	CGGCGC	exonic	EOMES	nonframeshift	4	2	0.33
chr11	111965548	111965551	ACTG	-	exonic	SDHD	frameshift	35	14	0.29
chr16	55844925	55844925	-	AC	intronic	CES1	.	28	16	0.36
chrX	109416689	109416689	T	-	UTR3	TMEM164	.	3	9	0.75
chr1	53793511	53793512	TG	-	exonic	LRP8	frameshift	9	4	0.31
chr1	54871567	54871570	CCGC	-	intronic	SSBP3	.	3	6	0.67
chr2	102314633	102314633	-	GGCAGC	intronic	MAP4K4	.	0	39	1.00
chr3	40557308	40557308	T	-	intronic	ZNF620	.	20	17	0.46
chr11	123455082	123455082	-	GGGAGG	intronic	GRAMD1B	.	0	31	1.00
chr17	1631341	1631343	GAG	-	exonic	WDR81	nonframeshift	9	9	0.50
chr3	167762678	167762678	-	A	intronic	GOLIM4	.	18	8	0.26
chr9	138648814	138648822	TCCCCACC	-	intronic	KCNT1	.	4	2	0.33
chr20	271204	271204	-	T	intronic	C20orf96	.	1	12	0.40
chr20	62369426	62369426	-	GGGGCG	intronic	LIME1	.	5	10	0.67

chr22	32340838	32340838	G	-	intronic	C22orf24,YV	.	6	4	0.40
chr1	54871567	54871570	CCGC	-	intronic	SSBP3	.	3	2	0.40
chr12	9572788	9572788	-	GCACCTG	ncRNA_exoni	DDX12P	.	0	31	1.00
chr12	88525006	88525006	A	-	intronic	CEP290	.	19	7	0.27
chrX	109416689	109416689	T	-	UTR3	TMEM164	.	8	4	0.29
chr1	200080462	200080462	-	T	intronic	NR5A2	.	15	25	0.63
chr6	32470636	32470636	-	T	intergenic	HLA-DRA,HL	.	7	4	0.31
chr9	43121655	43121658	TAAG	-	exonic	ANKRD20A2	frameshift	7	3	0.30
chr11	65359237	65359237	-	C	intronic	EHBP1L1	.	9	5	0.36
chr11	133789018	133789018	-	GGGGGT	intronic	IGSF9B	.	2	9	0.69
chr5	157236754	157236756	TCT	-	exonic	CLINT1	nonframeshift	93	32	0.26
chr10	124740240	124740249	GCGGGGC(	-	intronic	PSTK	.	0	6	1.00
chr11	102587154	102587154	-	A	intronic	MMP8	.	1	4	0.80
chr1	54871567	54871570	CCGC	-	intronic	SSBP3	.	6	3	0.33
chr3	184100961	184100961	-	T	intronic	CHRD	.	5	4	0.44
chr8	86574438	86574439	AC	-	ncRNA_exoni	REXO1L2P	.	6	3	0.33
chr16	88593211	88593211	T	-	intronic	ZFPM1	.	15	8	0.35
chr3	40557308	40557308	T	-	intronic	ZNF620	.	11	8	0.42
chr7	38810880	38810880	-	A	intronic	VPS41	.	14	18	0.51
chr17	79419160	79419185	GGGAGGG	-	intronic	BAHCC1	.	6	5	0.45
chr20	3063587	3063587	-	C	intronic	AVP	.	2	3	0.60
chr1	200080462	200080462	-	T	intronic	NR5A2	.	26	30	0.54
chr2	99439264	99439264	G	-	exonic	KIAA1211L	frameshift	4	6	0.60
chr8	68062183	68062184	TT	-	intronic	CSPP1	.	12	4	0.25
chr15	65209718	65209718	-	TGTC	intronic	ANKDD1A	.	8	3	0.27
chr19	42797295	42797296	AG	-	exonic	CIC	frameshift	10	19	0.66
